# Inhibition of inflammation and senescence with azole compound C7 enables expansion of hematopoietic stem and progenitor cells

**DOI:** 10.1093/stcltm/szag037

**Published:** 2026-07-01

**Authors:** Ze Hui Kok, Lin Ming Lee, Kelvin Y H Liu, John Ouyang, Vikneswari Rajasegaran, Samantha P S Lim, Jui Wan Loh, Jing Yi Lee, Abner H Lim, Cedric Chuan Young Ng, Jason Yongsheng Chan, Sudipto Bari, William Ying Khee Hwang

**Affiliations:** Division of Medical Sciences, National Cancer Centre Singapore, Singapore 168583, Singapore; Division of Medical Sciences, National Cancer Centre Singapore, Singapore 168583, Singapore; Division of Medical Sciences, National Cancer Centre Singapore, Singapore 168583, Singapore; Centre for Computational Biology, Duke-NUS Medical School, Singapore 169857, Singapore; Division of Medical Sciences, National Cancer Centre Singapore, Singapore 168583, Singapore; Division of Medical Sciences, National Cancer Centre Singapore, Singapore 168583, Singapore; Cancer Discovery Hub, National Cancer Centre Singapore, Singapore 168583, Singapore; Division of Medical Sciences, National Cancer Centre Singapore, Singapore 168583, Singapore; Division of Medical Sciences, National Cancer Centre Singapore, Singapore 168583, Singapore; Division of Medical Sciences, National Cancer Centre Singapore, Singapore 168583, Singapore; Cancer Discovery Hub, National Cancer Centre Singapore, Singapore 168583, Singapore; SingHealth Duke-NUS Blood Cancer Centre, Singapore 168583, Singapore; Division of Medical Sciences, National Cancer Centre Singapore, Singapore 168583, Singapore; Advanced Cell Therapy and Research Institute, Singapore 168583, Singapore; Division of Medical Sciences, National Cancer Centre Singapore, Singapore 168583, Singapore; SingHealth Duke-NUS Blood Cancer Centre, Singapore 168583, Singapore; Department of Haematology, Singapore General Hospital, Singapore 169856, Singapore; Cancer and Stem Cell Biology Programme, Duke-NUS Medical School, Singapore 169857, Singapore; SingHealth Duke-NUS Regenerative Medicine Institute of Singapore, Singapore 169857, Singapore

**Keywords:** cord blood, hematopoietic stem cell transplantation, cellular proliferation, cellular senescence, inflammation

## Abstract

Human hematopoietic stem and progenitor cells (HSPCs) derived from umbilical cord blood (UCB) are often insufficient in quantity for transplantation, necessitating efforts for *ex vivo* expansion. We previously developed a novel azole-based small molecule, C7, as a structural analog of the p38 mitogen-activated protein kinase inhibitor SB203580. C7 mediated robust *ex vivo* expansion of UCB HSPCs from unselected mononuclear cells, compared to its structural analogs and other published compounds. However, the molecular underpinnings of C7’s potency remained unclear. In this work, we demonstrate that C7 preferentially enhances the proliferation and viability of early HSPC subsets upon direct culture with pre-isolated HSPCs, while limiting mitochondrially-derived reactive oxygen species production linked to oxidative stress. C7 treatment enhances hematopoietic stem cell self-renewal and *in vivo* multilineage reconstitution without T- or myeloid lineage bias. Unlike existing p38 inhibitors, C7 targets multiple kinases and pathways to achieve superior HSPC expansion through suppression of NFκB and IL-10 inflammatory signaling and attenuation of stress/senescence‑associated markers (eg, SERPINB2 and CDKN1A/p21). This mechanism of action of C7 is distinct from those of other existing compounds used for *ex vivo* HSPC expansion and has implications for application in other *ex vivo* culture platforms and clinical settings.

## Graphical Abstract

**Graphical Abstract:**
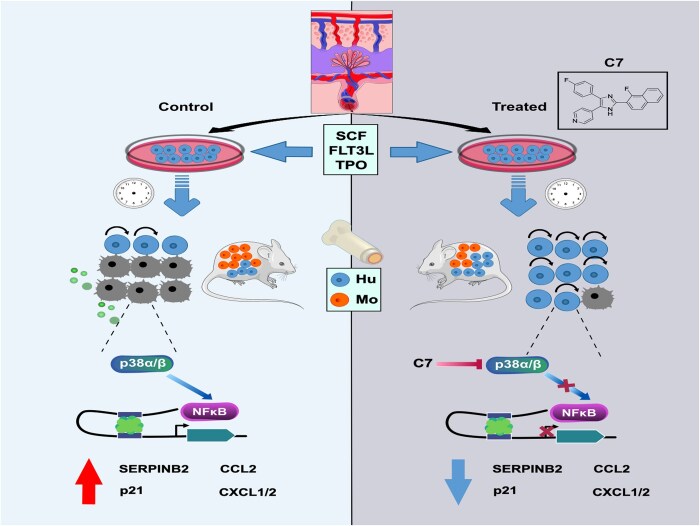

Significance Statement We have uncovered previously uncharacterized mechanisms of action of C7, a novel azole-based small molecule for enhanced *ex vivo* expansion of cord blood-derived hematopoietic stem and progenitor cells (HSPCs). We identify C7 as an inhibitor of additional protein kinase targets beyond its role as a p38 mitogen-activated protein kinase (MAPK) inhibitor, and as an agent mediating inhibition of inflammatory and senescence-related processes. This work positions C7 as a means of maximizing cord blood unit utilization for hematopoietic transplantation, and potentially as a means of anti-aging and regenerative therapies.

## Introduction

Hematopoietic stem cell transplantation (HSCT) is widely used as a means to reconstitute a healthy hematopoietic system in patients with blood disorders. These include hematopoietic malignancies (such as leukemias and lymphomas), and other non-cancerous conditions (such as autoimmune diseases and anemia).^1–4^ In particular, umbilical cord blood (UCB)-derived HSCs have the advantage of being more permissive to human leukocyte antigen (HLA) mismatches between donors and recipients, and therefore a lower incidence of graft-versus-host disease (GvHD).^1^^,^^5^ Unfortunately, UCB units often contain insufficient numbers of HSCs for the engraftment and reconstitution of adult recipients, limiting their use.

Recent strategies to obtain larger numbers of donor HSCs for transplantation have involved their *ex vivo* expansion using various small molecules (nicotinamide riboside, UM171, SR1) in cytokine-containing media, or polymer cultures prior to harvest and reinfusion.^1–3^^,^^6^ Several mechanisms by which these methods prevent the differentiation of HSCs while enhancing proliferation and long-term repopulation capacity have been described, including the inhibition of specific receptors involved in cellular signaling or epigenetic factors.^7–11^ However, with the possible exception of polymer-based cultures, few of these mechanisms have been linked to the suppression of various forms of stress which lead to reduced HSC function during *ex vivo* culture, including the production of reactive oxygen species (ROS), inflammation, and senescence.^12–14^

p38 Mitogen-Activated Protein Kinases (p38-MAPKs) regulate cellular responses to various forms of stress,^15^ and have been implicated in reactive oxygen species (ROS)-induced defects in HSC self-renewal and *in vivo* repopulating capacity.^16^ Pharmacological (SB203580) or genetic (shRNA) inhibition of p38 MAPKs have been shown to result in modest expansion of CD133^+^CD38^-^ HSCs or CD34^+^ HSPCs from UCB, respectively.^17^^,^^18^ We previously investigated the utility of a novel azole-based small molecule, C7, as a cost-effective and scalable means for *ex vivo* HSC expansion.^19^ C7 was derived from structure-activity-relationship (SAR) studies of SB203580, a p38α-MAPK inhibitor. We demonstrated that C7 treatment resulted in significant expansion of rare CD34^+^CD38^-^CD45RA^-^CD90^+^CD49f^+^ HSCs from UCB-derived mononuclear cells (MNCs), over the course of 10 days. In addition, C7 treatment of UCB MNCs enhanced hematopoietic stem and progenitor cell (HSPC) engraftment and long-term multilineage chimerism in immunodeficient mice compared to DMSO-only controls in BM and PB, and maintained the capacity for engraftment in secondary recipients. Yet, the cellular and molecular mechanisms by which C7 exerts its effects remain unclear.

In this current work, we demonstrate a direct effect of C7 on human UCB-derived CD34^+^ HSPC expansion, using *ex vivo* culture and *in vivo* transplantation assays. While comparative kinase inhibition profiling approaches established additional targets of C7, such as TGFβRI/II and CK1δ, single-cell transcriptomic approaches demonstrated that C7 inhibits several pro-inflammatory signaling pathways, including those involving NFкB and IL-10, as well as senescence-related transcriptional changes, which we have validated experimentally. Collectively, our results highlight the potential of C7 to perturb multiple targets beyond its canonical role as an inhibitor of p38 MAPK.

## Materials and methods

### Collection and isolation of UCB, PB and BM-derived CD34^+^ HSPCs

UCB units were obtained through the Singapore Cord Blood Bank (SCBB) as de-identified research-consented units unsuitable for public clinical banking. Usage of the UCB units was approved by the Research Advisory Ethics Committee of the SCBB (Ref. No. RP2104), the Centralised Institutional Review Boards of SingHealth (CIRB-SH, Ref. No. 2021-2346) and National Cancer Centre Singapore. Mononucleated cells (MNC) were first isolated from fresh UCB by density gradient centrifugation using Lymphoprep (STEMCELL Technologies, Canada; #18061). Fresh cord blood was diluted 1.5× with PBS, then layered onto Lymphoprep in SepMate Tubes (STEMCELL Technologies, Canada; #18061) and centrifuged at 1200 g for 10 minutes to obtain MNCs. Cells were then washed with PBS prior to immunomagnetic positive selection of CD34^+^ cells using EasySep Human CD34 Positive Selection Cocktail (STEMCELL Technologies, Canada; #18096C) according to the manufacturer’s instructions. Post-isolation purity was verified by flow cytometry to be >85%. Selected UCB-CD34^+^ Cells were then cryopreserved in 90% vol/vol autologous plasma (Thermo Fisher Scientific, United States; #368632) containing 10% v/v DMSO (Sigma Aldrich, United States; D2438) for future experiments. mPB- and BM-derived CD34^+^ (Lonza Group AG, Switzerland; 4Y-101C & 2M-101C) were procured commercially and cryopreserved prior to use.

### CD34^+^ HSPC culture, harvest, and counting

Cryopreserved UCB-CD34^+^ cells were thawed rapidly at 37 °C and pooled together (*n *> 30 donors per pool, unless otherwise stated) into pre-warmed StemSpan Animal Origin Free Media (StemSpan-AOF) (STEMCELL Technologies, Canada; #100-0130) supplemented with 50 ng/ml FLT-3 Ligand (FLT3L) (PeproTech, United States; #AF-300-19-100), 100 ng/ml thrombopoietin (TPO) (PeproTech, United States: #AF-300-18-100), and 100 ng/ml stem cell factor (SCF) (Peprotech, United States; #AF-300-07-100). Cryovials were thawed rapidly in a 37 °C water bath until minimal ice remained, then diluted dropwise into pre‑warmed medium and centrifuged to remove DMSO before culture initiation. Total nucleated cells were counted manually using a hemocytometer with trypan blue exclusion to determine viability. Cells were cultured at an empirically determined initial density of 1.0 × 10^5^/ml unless otherwise stated and maintained in a humidified, 5% CO_2_ incubator at 37 °C for up to 11 days. Where indicated, C7 and/or UM171 (STEMCELL Technologies, Canada; #72914) compounds were added at 5 μM and 50 nM respectively as performed previously,^19^ with DMSO used as a negative control. The DMSO (vehicle) concentration was matched across all arms (0.1% w/v). Respective media and compound replenishment of 1:1 was conducted on day 7. At the indicated time-points, cells were resuspended in PBS + 2% FBS (Cytiva, United States; #SV30160.03) and flow cytometry was performed using antibodies listed in Table S1A. Single stain controls consisting of CD34^+^ cells incubated with individual antibodies were used for fluorochrome compensation with Ultracomp eBeads Compensation Beads (Thermo Fisher Scientific, United States, 1222242). For 7-AAD staining, a mixture of equal quantities of live and dead UCB-HSPCs killed with 70% ethanol were used as a compensation control. Auto-compensation and data analysis were subsequently performed with FlowJo v10 software (FlowJo LLC, United States). Expansion fold was calculated based on manual cell counts obtained with Trypan blue staining, multiplied by percentages of desired cell populations from flow cytometry analyses and normalized relative to day 0 unexpanded cells. Single donor UCB, PB and BM-derived CD34^+^ cells were cultured and treated under similar conditions.

### CellTrace violet proliferation assay

Upon thawing, UCB-CD34^+^ cells were incubated with 6 μM CellTrace Violet/10^6^ cells for 20 minutes at 37 °C in the dark followed by incubation with 5 ml StemSpan AOF + 2% FBS (∼5× staining volume) as per the manufacturer’s protocol (Thermofisher, United States; C34557). Cells were then pelleted and resuspended in fresh StemSpan-AOF and cytokine cocktail with 5 μM C7 or DMSO control prior to flow cytometry at the indicated time-points.

### Mitochondrial function assays

UCB-MNCs were expanded with C7 or DMSO for 3 days before flow cytometry analysis. Cells were incubated with 0.5 μM MitoSOX Red (ThermoFisher, United States; M36007) in PBS at 37 °C with 5% CO_2_ for 30 minutes in the dark. Subsequently, cells were washed in PBS and stained with 7-AAD (Beckman Coulter, United States; IM3422) and CD45-PE-Cy7 (BD Biosciences, United States; 557748) prior to analysis on a BD LSRFortessa (BD Biosciences, United States).

Similarly, JC-1 dye was diluted to 2.5 μg/ml in PBS + 2% FBS during staining. Cells were incubated at 37 °C in 5% CO_2_ for 10 minutes in the dark prior to flow analysis.

### Colony forming unit (CFU) assay

Freshly-thawed, unexpanded UCB-derived CD34^+^ HSPCs, or HSPCs expanded with C7 or DMSO for 11 days, were suspended in Iscove’s Modified Dulbecco’s Medium (IMDM; Sigma-Aldrich, United States; I3390) supplemented with 2% FBS. Cells were then plated onto 35 mm dishes (Corning, United States; 351008) in MethoCult H4034 Optimum CFU media (STEMCELL Technologies, Canada; #04034) at empirically optimized densities of 500, 200, 100 or 50 cells per dish in duplicate in a humidified, 5% carbon dioxide (CO_2_) incubator at 37 °C. After 14 days, enumeration of colony-forming unit-granulocyte-macrophage (CFU-GM), colony-forming unit-granulocyte-erythroid-macrophage-megakaryocyte (CFU-GEMM), and burst-forming unit-erythroid (BFU-E) units was performed. Expansion fold was calculated based on the number of colonies formed out of the total cell numbers at day 11 relative to that using uncultured cells plated on day 0 of culture.

### Animal experiments

Experimental protocols were approved and performed in accordance with the recommendations by the SingHealth Institutional Animal Care and Use Committee (IACUC Ref. No. 2021/SHS/1654). NOD.Cg-Prkdc^scid^ Il2rg^tm1Wjl^/SzJ, also known as non-obese diabetic (NOD)–severe combined immunodeficient (SCID) gamma (NSG) mice, were purchased from Jackson Laboratory (Bar Harbor, United States) and housed in cages of 5 of the same gender in SingHealth Experimental Medicine Centre (SEMC). Sterilized food and water were accessible ad libitum. Following acclimation and successful breeding for 3 generations, the sub-lethally irradiated (240 cGy) 8-12 week old primary recipient mice were randomly divided into 4 experimental groups for tail vein administration of: (a) DPBS; (b) unexpanded UCB–CD34^+^; (c) DMSO-expanded UCB–CD34^+^; and (d) C7- expanded UCB–CD34^+^. Prior to any cell injections, all NSG mice received prophylactic antibiotics (0.1 mM neomycin and 0.1 mM polymyxin B in autoclaved acidified ddH_2_O of pH 2.2) and immunosuppressive drugs (10-15 mg/kg cyclosporin injections via intraperitoneal injection) over 3 weeks to minimize bacterial infection and reduce chances of graft vs. host disease (GvHD) respectively.

To model a clinically relevant “expand‑then‑infuse” scenario, unexpanded CD34^+^ cells were transplanted immediately after thaw at 250, 2500, or 25 000 cells per mouse. For expanded conditions, cultures were initiated with 250, 2500, or 25 000 input CD34^+^ cells and expanded for 11 days; the entire harvested culture output derived from each respective input dose was transplanted per mouse. For transparency, the total viable nucleated and CD34^+^ cell numbers infused for expanded arms are reported in Table S2. Submandibular bleeding of mice was conducted at weeks 3, 6, 10 and 13 after injection followed by flow analysis to observe engraftment of human cells. Mice were then sacrificed at week 16 for bone marrow harvest. Harvested cells were stained for flow cytometry using antibodies listed in Table S1B.

For secondary transplantation experiments, bone marrow cells from each treatment group were pooled together regardless of initial injected cell dosages. hCD45^+^ cells were magnetically isolated using CD45 microbeads (Miltenyi, United States; 130-045-801) according to the manufacturer’s protocol. Selected hCD45^+^ cells were then cryopreserved in 90% FBS and 10% DMSO prior to subsequent secondary engraftment experiments. Secondary engraftment was conducted by resuspending freshly thawed hCD45^+^ cells in 95% PBS and 5% FBS to be injected into irradiated mice. 2 × 10^6^ hCD45^+^ cells were injected into each mouse for every treatment group. Engraftment levels for each treatment group was observed at similar time points as primary engraftment: submandibular bleeds at weeks 3, 6, 10, and 13 followed by sacrifice and bone marrow harvest at week 17.

### KINOMEscan and BioMAP diversity plus profiling

Details of the above are provided in Supplemental Methods.

### Western blot

UCB-CD34^+^ cells were lysed in 1X RIPA Buffer (Cell Signalling Technology, United States; #9806), supplemented with cOmplete mini EDTA-free Protease Inhibitor Cocktail (Roche Diagnostics, Germany; #4693124001) and PhosStop (Roche Diagnostics, Germany; #4906837001). Protein quantification was then conducted using DC Protein assay with BSA Standards (Bio-Rad Laboratories, United States; #5000112) according to the manufacturer’s instructions. Equal amounts of protein were resolved on SDS-PAGE gels and transferred onto methanol-activated 0.2 μm PVDF membranes via Trans-blot Turbo Mini System (Bio-Rad Laboratories, United States). Membranes were then blocked with 5% BSA in 0.1% TBST, followed by incubation with primary and secondary antibodies listed in Table S1C. Immunoblots were developed using ECL Prime Western Blotting System (Cytiva, United States; RPN2232) or SuperSignal West Femto (Thermo Scientific, United States; 34096) and detected digitally with the Chemidoc XRS+ Imaging System (Bio-Rad Laboratories, United States). Quantifications were then performed using ImageJ (National Institutes of Health, United States) software.

### RNA extraction and qRT-PCR

RNA extraction of UCB-CD34^+^ cells was conducted with Qiagen RNeasy Plus Micro Kit (Qiagen, United States; 74034) as per the manufacturer’s instructions and quantified with Nanodrop (Thermo Fisher, United States). 1ug of RNA was then converted into cDNA with the iScript cDNA Synthesis Kit (Bio-rad Laboratories, United States; 1708891) according to the manufacturer’s protocol. Quantitative real-time reverse transcription polymerase chain reaction (qRT-PCR) was performed using the Bio-rad CFX96 system with SsoFast Evagreen Supermix (Bio-rad Laboratories, United States; 1725201) and 0.1ul of each primer (100 nM concentration) according to the manufacturer’s instructions (refer to Table S3 for list of qPCR primers used). The qRT-PCR protocol involves enzyme activation at 95 °C for 3 minutes, followed by 35 cycles of denaturation at 95 °C for 10 seconds and annealing/extension at 60 °C for 5 seconds.

### Cholesterol assay

Details of the above are provided in Supplemental Methods.

### Senescence-associated β-galactosidase (SA-β‑gal) activity assay

SA‑β‑gal activity was assessed using the CellEvent Senescence Green Flow Cytometry Assay (Invitrogen, United States; C10841) with FITC settings. Cells were stained with surface markers (CD34‑PE and CD38‑APC) prior to fixation with 0.5% paraformaldehyde (PFA) for 10 minutes at room temperature in the dark. Cells were then washed and incubated with Senescence Green probe (1:100 in assay buffer, 2 hours at 37 °C in the dark without CO_2_). Compensation was established using single‑stained controls for each fluorophore (including probe‑only controls for the Senescence Green signal), and gates were set using fluorescence‑minus‑one (FMO) controls lacking the Senescence Green probe. SA‑β‑gal positivity was quantified within the CD34^BR^ CD38^−^ gate.

### Enzyme-linked immunosorbent assay (ELISA)

Cell cultures consisting of C7 or DMSO-treated UCB-CD34^+^ HSPCs were harvested at the indicated time-points and centrifuged at 450 g for 5 minutes. For each treatment and time-point, 100 μl of undiluted conditioned media was transferred to each well of an ELISA strip (Human Cytokine ELISA Plate Array II, Signosis, United States; EA-4010) coated individually with antibodies targeting 8 cytokines. Samples were incubated overnight at 4 °C, followed by biotin-labeled secondary antibodies, streptavidin-HRP, and substrate (2 hours, 1 hour, and 1 hour respectively at room temperature), with wash steps in between incubations according to the manufacturer’s instructions. Thereafter, the optical density was determined at 450 nm using Tecan Spark Cyto (Tecan, Switzerland) then normalized against media-only samples as a negative control.

### scRNA-seq sample preparation, sequencing, data processing and analysis

Details of the above are provided in Supplemental Methods.

### Gene set enrichment analysis (GSEA)

To avoid selective interpretation, we performed DE and pathway enrichment across all pairwise contrasts among the 4 treatment conditions (A–D) at each timepoint, and report the full results as Supplemental Table S4 (DEGs) and Table S5 (GSEA). Single HSC transcriptomes were pseudobulked and analyzed via GSEA via the clusterProfiler package (v4.12.0).^20^ mSigDB (v2024.1.Hs)^21^ hallmark (H) gene sets (*n *= 50 gene sets), curated pathways from Biocarta, KEGG, PID, Reactome and wikipathways (mSigDB C2: CP set, *n *= 2953 pathways) and additional mSigDB genesets containing the gene *CYP1B1* (Search term: CYP1B1, *n *= 533 gene sets) were collated and used. The fast GSEA approach with 10000 permutations was performed. Normalized enrichment scores (NES) and FDR *q*-values were obtained from differential expression signatures comparing Group B (C7) vs. A (DMSO- only) for each time-point (Days 3, 7, and 11). FDR values < 0.05 were considered as significantly enriched. Analysis of leading edge gene overlaps was performed with GeneVenn (https://www.bioinformatics.org/gvenn).

### Statistical analysis

Data are presented as mean ± SD unless stated otherwise. “*n*” denotes technical replicates (eg, replicate wells/reads from the same biological sample) and “*N*” denotes biological replicates (independent donors/donor pools or independent mice, as applicable). Data normality was assessed using Shapiro–Wilk test prior to parametric testing; where normality was not assumed, non-parametric tests were used as indicated. For 2‑group comparisons, an unpaired 2‑tailed *t*‑test or multiple *t*-test was used for normally distributed data and Mann–Whitney U test otherwise. Statistical analyses were performed in GraphPad Prism v8.0 or later (GraphPad Software, Inc., United States).

## Results

### C7 mediates preferential expansion of early HSPCs

**Figure 1:**
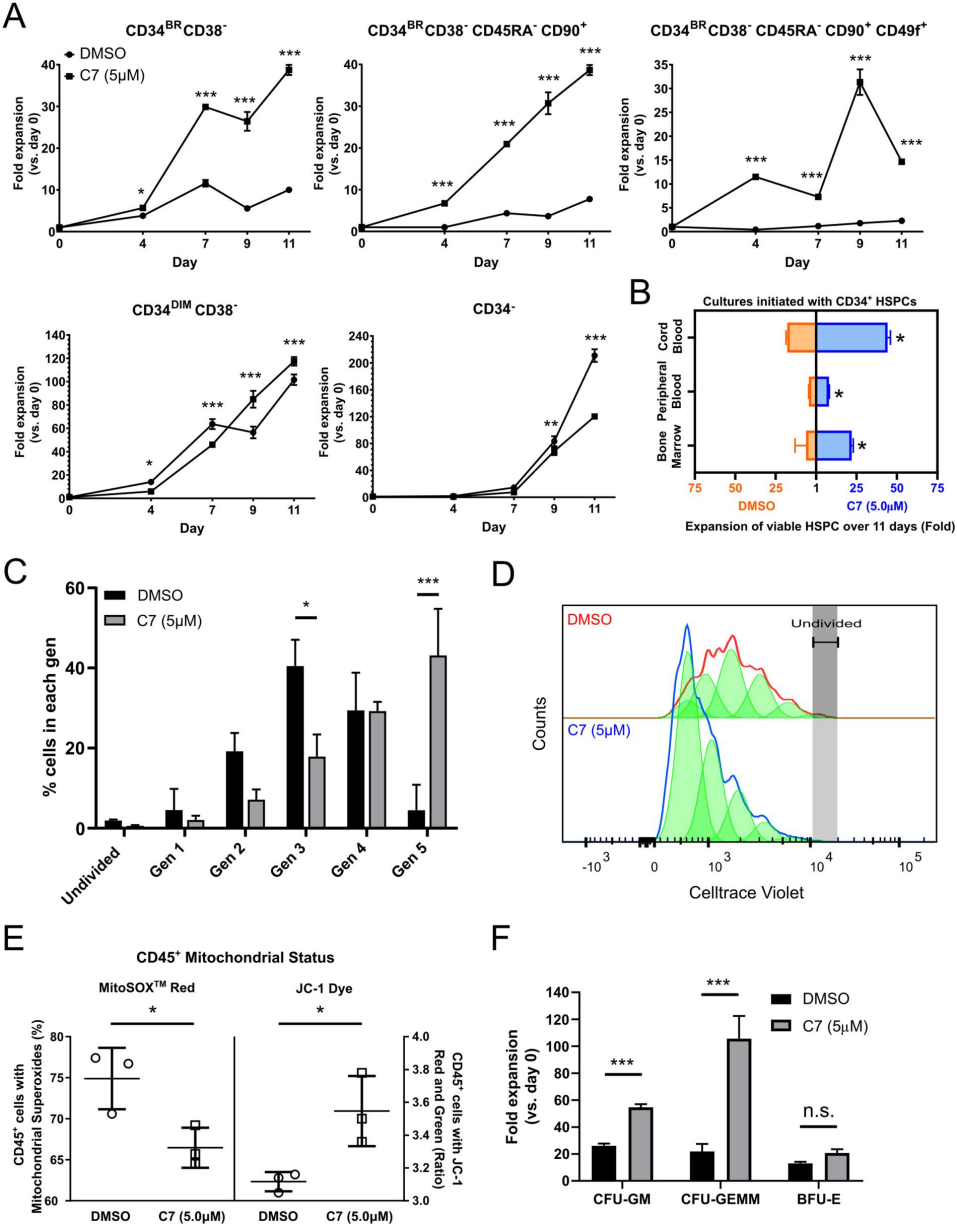
C7 drives preferential expansion of early as well as HSC-enriched HPCs. (A) Cell counts indicating fold expansion, relative to Day 0, of UCB CD34^+^ HSPCs from a pooled donor sample (*n* ≈ 30) cultured in Stemspan-AOF media containing hSCF, hFLT3L and hTPO, in the presence of C7 (5µM) or DMSO. A 1:1 replenishment of fresh media containing cytokines and compounds was performed on day 7. The data for each subset were calculated using flow cytometry-defined percentages with gating method indicated above each panel. Data represents mean ± SD of *n*=3 counts for 1 UCB donor. See also Figure S1A and B (results for 1 additional donor). (B) Fold expansion of C7 (5µM) or DMSO-treated HSPCs from BM, PB or UCB (mean ± SD for *N*=3 donors each) at Day 11, relative to Day 0. Cells were cultured in Stemspan-AOF media with cytokines and compounds as in (A), with media replenishment on Day 7. (C) Celltrace assay depicting proportions of C7 (5µM) or DMSO-treated UCB CD34^+^ HSPCs from 1 donor (mean ± SD for *n*=2 treatment replicates) in generations 0 (undivided) to 5 (corresponding to highest number of cell divisions). Cells were incubated briefly (20 minutes) in CellTrace Violet reagent followed by 11 days of culture in Stemspan-AOF media with cytokines and the respective compounds prior to flow cytometry measurements. (D) Representative flow cytometry analysis of Celltrace Violet staining for the experiment in (C), depicting increased cell division (higher proportion of negative-staining populations) for C7-(bottom) relative to DMSO (top)-treated cells. (E) Effect of C7 (5µM) or DMSO-only treatment on mitochondrial superoxide production (MitoSOX assay) and mitochondrial membrane potential (JC-1 dye) in UCB-derived CD45^+^ hematopoietic cells. Cells were cultured for 3 days in Stemspan-AOF media with cytokines plus the respective compounds prior to assays. Data represents mean ± SD *N*=3 donors per treatment. (F) CFU assay of UCB CD34^+^ cells expanded in Stemspan-AOF media with cytokines plus C7 (5µM) or DMSO for 11 days, followed by re-plating without compounds at 500 cells/dish in MethoCult for CFU assays. Enumeration of CFU-GM, CFU-GEMM and BFU-E colonies was performed at Day 14 post-replating. Data represents mean ± SD of *n*=4 experimental replicates for 1 donor. See also Figure S1C (results for 1 additional donor). *** indicates *P* ≤ .001,**P ≤ .01, *P ≤ .05 and n.s. (not significant) otherwise, for comparisons involving C7 vs. DMSO-only controls by multiple *t*-test. Abbreviations: HSC, hematopoietic stem cells; HPC: hematopoietic progenitor cells; HSPC: hematopoietic stem and progenitor cells; UCB: umbilical cord blood; BM: bone marrow; PB: peripheral blood; AOF: animal origin-free; SCF: stem cell factor; FLT3L: FLT-3 ligand; TPO: thrombopoietin; DMSO; dimethyl sulfoxide; CFU: colony-forming unit; GM: granulocyte-monocyte; GEMM: granulocyte-erythrocyte-monocyte-megakaryocyte; BFU: burst-forming unit-erythrocyte.

UCB-derived CD34^+^ cells were cultured for up to 11 days in the presence of C7 (5 µM) or DMSO controls. At Days 4, 7, 9 and 11, the cell counts and fold expansion of various HSPC subsets relative to Day 0 were determined by flow cytometry as follows: Early HPCs (CD34^bright^ CD38^-^), late HPCs (CD34^dim^CD38^-^), HSCs (CD34^bright^CD38^-^CD45RA^-^CD90^+^CD49f^+^), and differentiated CD34^-^ cells.^22^^,^^23^ C7 treatment resulted in significantly improved fold expansion of HSPC subsets, with the maximum differences between C7 and DMSO controls being observed on Day 11 for early HPCs (C7: 38.73 ± 1.22 vs. DMSO: 10.02 ± 0.45; *P *< .001), and on Day 9 for HSCs (C7: 31.34 ± 2.67 vs. DMSO: 1.78 ± 0.16; *P *< .001), whereas expansions of CD34^dim^ and differentiated HPCs were more similar to DMSO controls (Figure 1A; Figure S1A and B). In separate experiments, C7 also mediated superior expansion of CD34^+^ HSPCs derived from multiple sources (bone marrow, peripheral or cord blood) (Figure 1B).

To determine if the observed expansion was due to enhanced cellular proliferation or viability, we performed CellTrace dye dilution and mitochondrial function assays, the latter being a readout of early apoptosis. CellTrace assays revealed that by Day 11 of expansion, a greater proportion of C7-treated early HPCs exhibited fifth-generation doublings compared to DMSO controls (43.11% ± 11.65% vs. 4.49% ± 6.35%; *P *< .001), indicative of increased cell division (Figure 1C and D). Moreover, C7 treatment reduced the percentage of CD45^+^ hematopoietic cells producing mitochondrial superoxides (as measured by MitoSOX assay), while the mitochondrial membrane potential (measured using JC-1 dye) was increased (Figure 1E). To determine how C7 influences the differentiation status of HSPCs, we cultured CD34^+^ cells for 11 days in the presence of C7 or DMSO only, followed by colony-forming unit (CFU) assays. C7 treatment resulted in greater fold expansions of relatively primitive CFU-GEMM units (C7: 106.52 ± 16.78 vs. DMSO: 21.84 ± 5.58; *P *< .001) compared to CFU-GM or BFU-E, suggesting increased proliferation of early HPCs (Figure 1F; Figure S1C). Taken together, these results indicate that C7 treatment leads to the preferential expansion of early HSPCs, in part by improving cellular viability *ex vivo*, thus reinforcing its role as a robust agent for clinical HSPC expansion.

### C7 enabled superior HSPC engraftment and self-renewal in an immunodeficient mouse model

**Figure 2:**
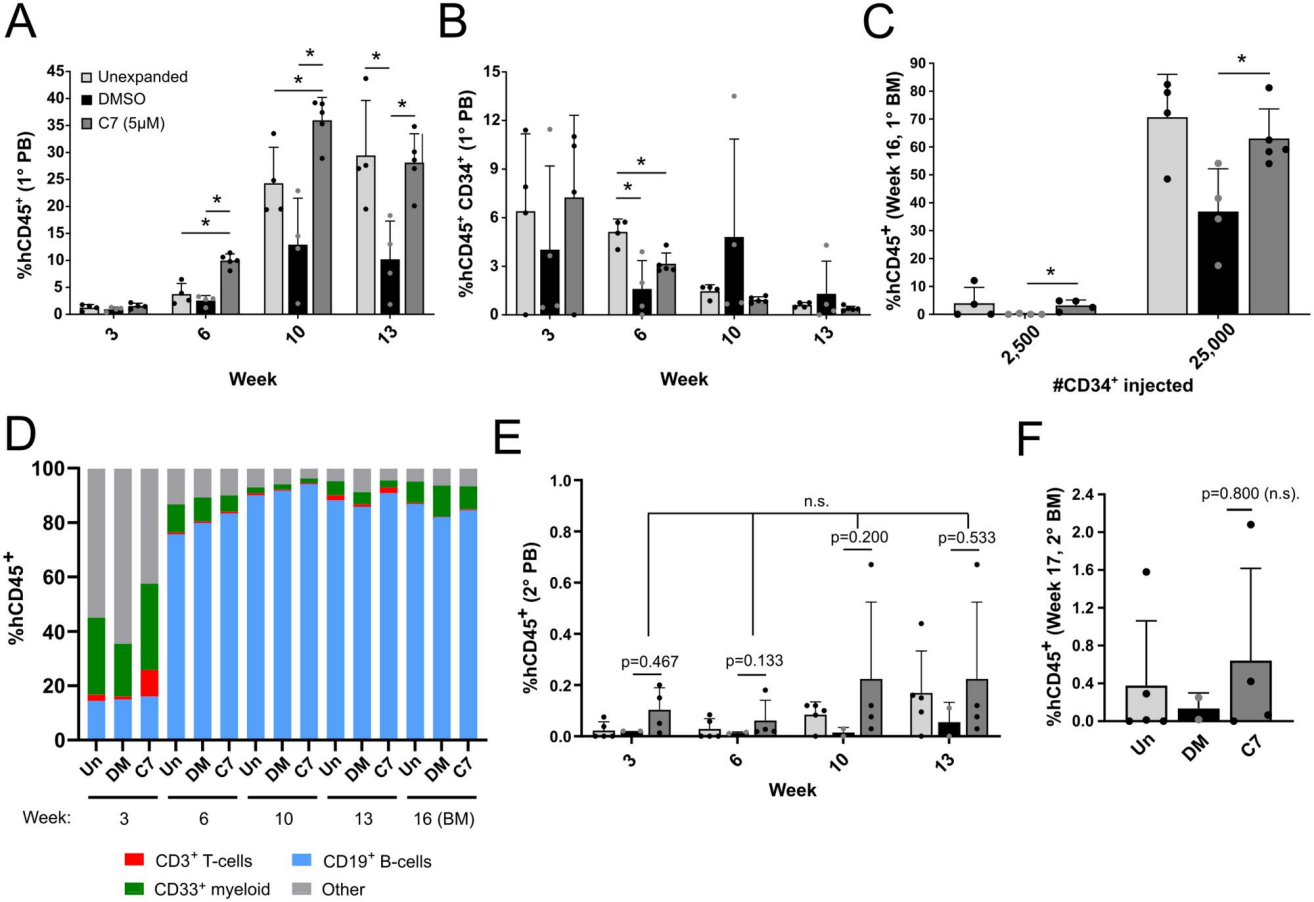
C7 enhances long-term multilineage engraftment and self-renewal of UCB HSPCs *in vivo*. (A) Flow cytometric analysis of hCD45^+^ engraftment in PB of primary NSG murine recipients, at Weeks 3-13 following transplantation of 25 000 freshly-thawed (unexpanded), DMSO or C7 (5µM)-treated UCB CD34^+^ HSPCs expanded for 11 days in Stemspan-AOF media with cytokines. For DMSO and C7 treatments, the entire output resulting from expansion of 25 000 starting HSPCs was used for transplantation. Submandibular bleeds were performed at the indicated time-points (Week 3-13) prior to flow analysis of PB. Data represent mean ± SD for *N* = 4-5 mice per treatment and time-point. See also Table S2 for details of numbers of total viable nucleated and CD34^+^ HSPCs transplanted per mouse and treatment group. (B) Levels of hCD45^+^ CD34^+^ HSPC engraftment as in (A), in PB of primary murine recipients. See also Figure S2A-C for detailed breakdown of PB engraftment data by lineage. (C) Levels of hCD45^+^ engraftment in BM harvests of primary murine recipients from (A) which were sacrificed at Week 16. Data for mice transplanted with 2 500 or 25 000 unexpanded HSPCs, or with the total outputs from the same numbers of starting HSPCs (DMSO and C7) are shown. See also Figure S2D-G for detailed breakdown of BM engraftment data by lineage. (D) Analysis of relative proportions of T- (CD3^+^), B- (CD19^+^), and myeloid lineage (CD33^+^) engraftment at the indicated time-points. Data is expressed as % of total hCD45^+^ from primary murine recipients transplanted with unexpanded, DMSO-only or C7-treated UCB CD34^+^ HSPCs. Un, unexpanded; DM, DMSO. (E) Levels of hCD45^+^ engraftment in PB of secondary murine recipients at Week 3-13 of transplantation. BM cells from each treatment (unexpanded, DMSO or C7) in (A) were pooled irrespective of initial cell dosages (2500 or 25 000 HSPCs) and hCD45^+^ cells were isolated by MACS. 2x10^6^ primary BM-derived hCD45^+^ cells were transplanted per secondary recipient. PB was harvested as in (A) at the indicated time-points (Week 3-13) for flow cytometric analysis of hCD45^+^ percentages. Data represents mean ± SD for *N*=2-5 secondary recipients per treatment group and time-point. (F) Levels of hCD45^+^ engraftment in BM harvests of secondary murine recipients at Week 17. Data represents mean ± SD for *N*=2-5 mice per treatment group. Un, unexpanded; DM, DMSO. *** indicates *P* ≤ .001,**P ≤ .01, *P ≤ .05 and n.s. (not significant) otherwise, for the indicated pairwise comparisons between unexpanded, C7 and DMSO-only controls by Mann-Whitney *U*-test. Abbreviations: UCB: umbilical cord blood; HSPC: hematopoietic stem and progenitor cells; PB: peripheral blood; BM: bone marrow; NSG: NOD-Scid-Gamma; DMSO: dimethyl sulfoxide; AOF: Animal origin-free; MACS: Magnetic-Activated Cell Sorting.

To assess the functional consequences of C7 treatment on early HPCs, we transplanted freshly thawed CB CD34^+^ progenitors unexpanded (Day 0) or expanded in the presence of C7 or DMSO for 11 days, into sub-lethally irradiated NOD-SCID-Gamma (NSG) mice. Unexpanded cells were injected at doses of 250, 2500, or 25 000 per mouse. For expanded cells, initial cultures of 250, 2500 or 25 000 cells were treated for 11 days with C7 or DMSO only, and the entire output from the respective expansions were used for transplantation. Peripheral blood (PB) analysis of hCD45^+^ levels up to Week 13 revealed superior engraftment of C7-expanded cells relative to DMSO controls (Dose 25 000 C7: 28.12% ± 5.34% vs. DMSO: 10.19% ± 7.08%; *P *= .016), comparable to unexpanded grafts (29.45% ± 10.19%) (Figure 2A). Similar trends, though less significant, were observed for the CD34^+^ compartment in PB up to Week 6 (Figure 2A), and in T- (CD3^+^), B-(CD19^+^) and myeloid cell (CD33^+^) fractions (Figure S2A-C). Improved chimerism (%hCD45^+^) in Week 16 bone marrow (BM) harvests was also observed with C7 treatment relative to DMSO-only (Dose 25 000 C7: 63.02% ± 10.60% vs. DMSO: 36.85% ± 15.29%; *P *= .032) (Figure 2C; Figure S2D-G). Overall, multiple lineages including T- (CD3^+^), B-(CD19^+^) and myeloid cells (CD33^+^) were detectable with C7 treatment at Week 3 of PB and thereafter, although B cells eventually pre-dominated in PB or BM (Figure 2D). Finally, secondary transplantation assays using hCD45^+^ cells isolated from the primary recipients demonstrated increased, although not statistically significant (*P *> .05), engraftment in PB (Week 3-13) and BM (Week 17) for C7 vs. DMSO-expanded cultures (Figure 2E and F). Thus, CD34^+^ HSPCs expanded *ex vivo* with C7 exhibited enhanced long-term hematopoietic potential, relative to DMSO-expanded cells.

### Kinase inhibition profiling and cell-based assays identify novel targets of C7

**Figure 3:**
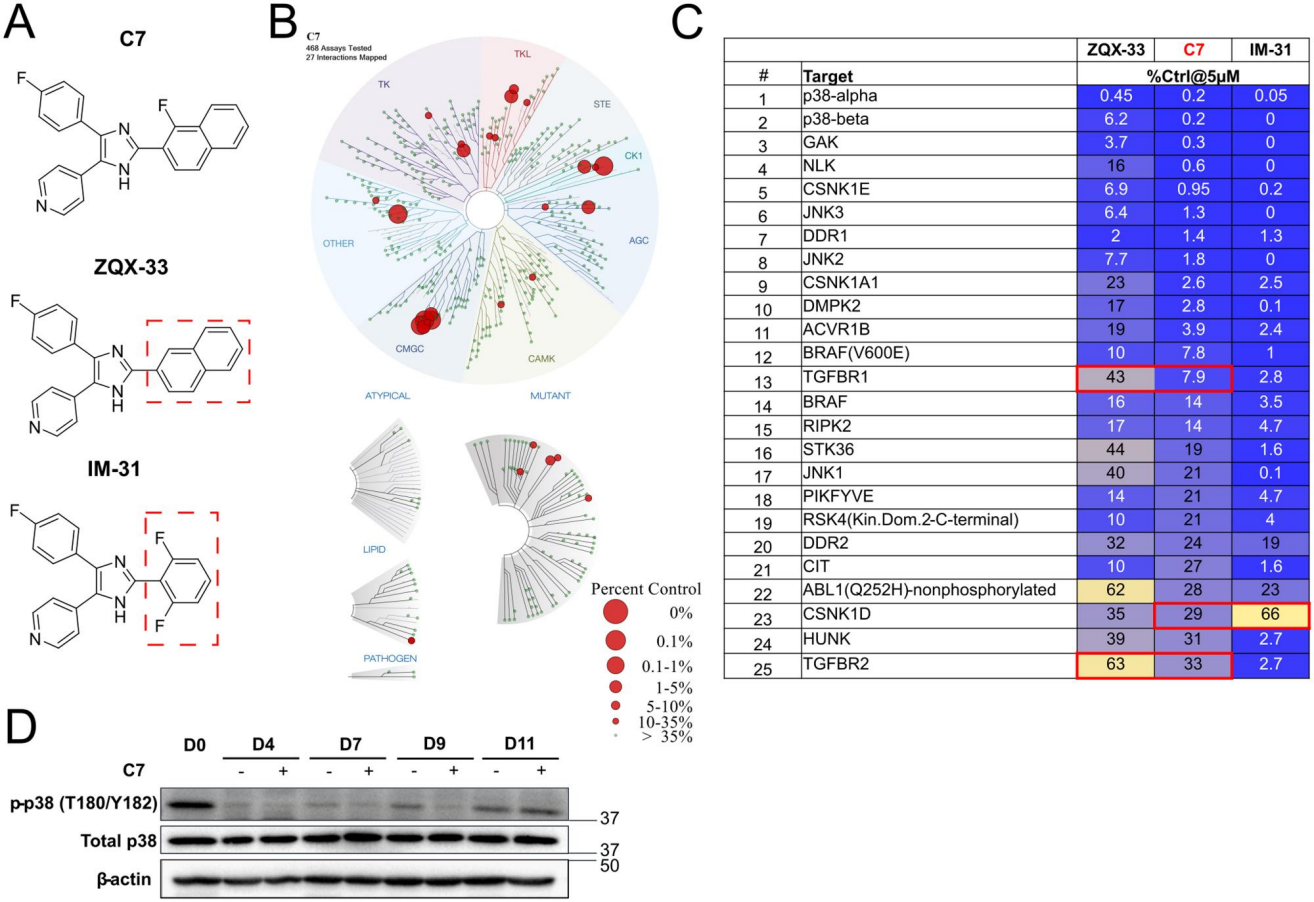
Kinase inhibition profiling identifies novel targets of C7. (A) Chemical structures of C7 as well as control compounds ZQX-33 and IM-31. Structural moieties differing from C7 are boxed in red. (B) TreeSPOT analysis of kinase targets inhibited by C7, based on the KinomeSCAN assay. Lower “percent control” indicates stronger degree of kinase-ligand binding inhibition mediated by C7 relative to negative controls (DMSO). See also Figure S3A, data for ZQX-33 and IM-31. (C) Top hits from KinomeSCAN Assay for C7, ZQX-33 and IM-31 profiled at 5μM each. The top 25 kinase targets for C7 are shown and ranked in order of decreasing inhibition by C7. Darker shading indicates stronger kinase inhibition by the respective compound relative to DMSO controls. Red boxes indicate comparisons of particular interest between C7 and ZQX-33 or IM-31. (D) Western blot analysis of phospho-p38 and p38 protein levels in UCB-CD34^+^ cells at Day 0 (unexpanded), and cells cultured in StemSpan-AOF media containing cytokines and C7 (5µM) or DMSO for the indicated time-points (D0-D11, day 0-day 11). Data represents 1 of 2 pooled samples from multiple donors. Abbreviations: DMSO: dimethyl sulfoxide; UCB: umbilical cord blood; AOF: Animal origin-free.

To establish specific molecular targets of C7, we conducted kinase activity inhibition profiling using the KinomeSCAN Assay Platform, which quantitatively measures the ability of test compounds to competitively inhibit binding interactions between individual kinases and their respective ligands. As reference controls, we profiled the C7 structural analogs ZQX-33 and IM-31 (previously C31 and C21, respectively),^19^ which contain modifications to the 1-fluoronapthalene moiety (Figure 3A), at the same concentration as C7 (5 µM). Both ZQX-33 and IM-31 were previously profiled to be about 2-fold less efficient at expansion of HSPCs compared to C7.^19^ TreeSPOT analysis revealed that C7 mediated significant inhibition of kinases belonging to several subfamilies, which were partially overlapping with those inhibited by ZQX-33 and IM-31 (eg, CMGC and CK1) (Figure 3B; Figure S3A). More specifically, KinomeSCAN profiling confirmed p38-MAPK (p38α and β) as the kinases which were most potently inhibited by C7 (0.2% of DMSO-treated controls) (Figure 3C). Among the top 25 kinases most strongly inhibited by C7, we identified several (TGFβRI/II, STK36, JNK1, and ABL1) as being more potently inhibited by C7 compared to ZQX-33, and CK1δ when compared against IM-31. However, none of these kinases were specifically inhibited by C7 relative to both ZQX-33 and IM-31, suggesting that all 3 compounds have unique binding activities. We also observed an increasing degree of inhibition for many kinase targets in the order ZQX-33 > C7 > IM-31, which might be correlated with the number of fluorine substituents. Western blot profiling confirmed inhibition of p38-MAPK activity (phospho-p38) by C7 across multiple time-points (Figure 3D).

We also employed BioMAP profiling of C7 (ZQX-45) at various concentrations (500 nM-15µM) to obtain further insights into its functional activities, and to predict safety and toxicity profiles (Figure S3B and Supplemental Methods). At our experimentally utilized concentration (5 µM), C7 treatment was associated with the altered activity of multiple cytokines involved in inflammation or immune functions, as well as factors involved in tissue remodeling (Figure S3C). C7 biological readouts were matched to 3 other known p38-MAPK inhibitors in the BioMAP assay library (Figure S3D), consistent with the KinomeSCAN results. Incidentally, the TGFβRI inhibitor SB431542 was identified when C7 was profiled at a higher dose of 15 µM, implying TGFβRI as a potential additional target of C7. Finally, predicted toxicity issues at 5 µM were mainly immunosuppression and skin irritation, but not more severe effects on other organs (Figure S3E). In all, kinase inhibition assays and *in vitro* modeling suggested that C7 may modulate signaling pathways or processes other than just the p38-MAPK pathway, with minimal toxicity.

### Single-cell transcriptomic profiling identifies C7 as an inhibitor of inflammation and senescence

**Figure 4:**
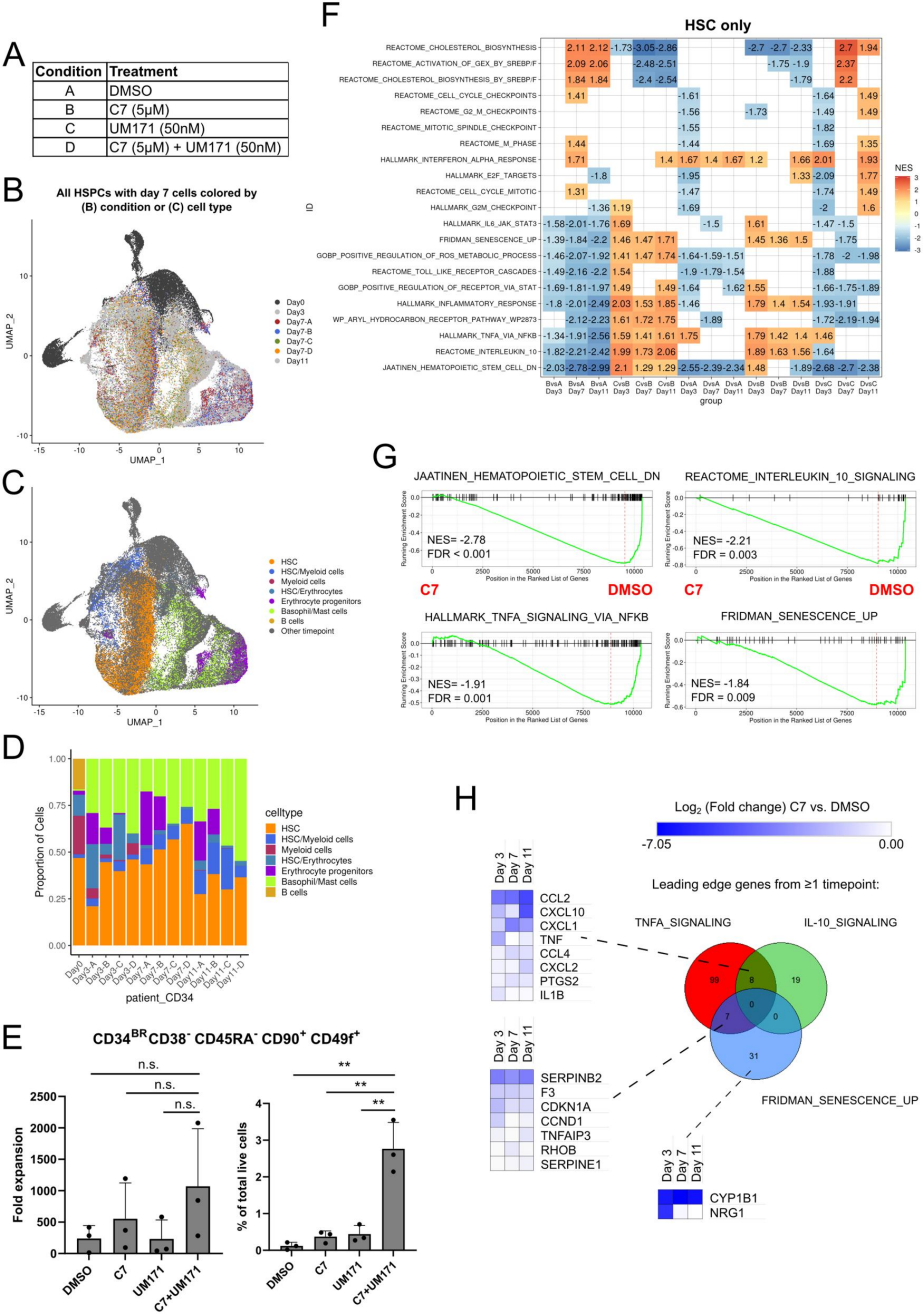
C7 inhibits transcriptional changes involving inflammation and senescence during *ex vivo* HSPC expansion. (A) List of treatment groups and their abbreviations (A-D) used for scRNA-seq experiment involving UCB CD34^+^ HSPCs. (B) UMAP plots of all HSPCs colored by treatment (A-D) for Day 7. HSPCs from Day 0 or other timepoints are colored in black and grey, respectively. Data is representative of *N*=3 donors per treatment group and time-point. See also Figure S4A, coloring by treatment for Day 3 and Day 11-specific HSPCs. (C) UMAP plots of all HSPCs colored by cell type for Day 7. HSPCs from other timepoints are colored in grey. See also Figure S4B, coloring by cell type for Day 3 and Day 11-specific HSPCs. (D) Cell proportion plot quantifying the relative abundances of each cell type within CD34^+^ HSPC cultures, for each day and treatment.Values are plotted as fractions of total cells, 0-1. (E) Fold expansion relative to Day 0 (left panel) and percentages (right panel, out of 7-AAD^-^ live cells) of HSC fractions (CD34^BR^ CD38^-^ CD45RA^-^ CD90^+^ CD49f^+^) derived from starting UCB-CD34^+^ HSPCs expanded in StemSpan-AOF with cytokines for 7 days with C7 (5µM) alone or in combination with or UM171 (50nM), with DMSO as control. Data represent mean ± SD for *N*=3 donors. See also Figure S5A and b for corresponding data at Day 3 and Day 11, and for early HPC (CD34^BR^ CD38^-^) and CD90^+^ fractions. (F) Heatmap detailing Normalized Enrichment Scores (NES) for pairwise gene set enrichment analysis (GSEA) comparisons of interest among the 4 treatment conditions (A-D). Gene sets attaining statistical significance of FDR < 0.05 are shown. Positive NES scores (processes up-regulated in first vs. second condition) are highlighted in orange, whereas negative NES scores (down-regulated in first vs. second condition) are indicated in blue. (G) GSEA enrichment plots for selected processes down-regulated in C7 vs. DMSO controls (negative enrichment scores). (H) Venn diagram showing overlap among leading edge genes contributing to negative enrichments for the indicated gene sets. For each gene set, leading edge genes were combined across 3 time-points and used for the analysis. The heatmaps indicate Log_2_-fold changes (C7 vs. DMSO) of selected genes according to timepoint. Genes were ranked and listed by their average fold change across the 3 time-points and those among the most strongly inhibited are shown. *** indicates *P* ≤ .001,**P ≤ .01, *P ≤ .05 and n.s. (not significant) otherwise, for the indicated pairwise comparisons between treatment groups by unpaired 2-tailed *t*-test. Abbreviations: HSPC, hematopoietic stem and progenitor cells; HPC, hematopoietic progenitor cells; UCB, umbilical cord blood, scRNA-Seq, single-cell RNA-Seq; UMAP, Uniform Manifold Approximation and Projection; AOF, Animal Origin-free; DMSO, dimethyl sulfoxide; NES, Normalized Enrichment Scores; GSEA, Gene Set Enrichment Analysis; FDR, False Discovery Rate.

To gain further insights as to C7’s mechanism(s) of action leading to HSPC expansion, we performed single-cell RNA-Seq (scRNA-Seq) on UCB-CD34^+^ cells cultured for 3, 7 or 11 days with (A) DMSO only or (B) C7 (5 µM). We also explored the potential for C7 to demonstrate either distinct or combinatorial effects with the previously characterized small molecule UM171^24^ in enhancing HSPC expansion, by including 2 additional treatments: (C) UM171 (50 nM), or (D) C7 plus UM171 (Figure 4A). Following UMAP dimensionality reduction, we performed clustering analysis of 72 151 unexpanded (Day 0) and treated HSPCs (Conditions A-D as above, on days 3, 7 and 11 of treatment) (Figure 4B; Figure S4A). We observed distinct segregation of treated HSPCs from untreated (Day 0) cells, indicative of transcriptional effects. Next, we cross-referenced the data against 7 defined cell types corresponding to HSCs or differentiated lineages based on gene expression markers as detailed in previous studies^25^^,^^26^ (Figure 4C; Figure S4B and C). At Day 7, whereas cells from all 4 treatments were represented within the HSC cluster, UM171-only and combination-treated cells (Conditions C & D) occupied a distinct subset of the basophil//mast cell fraction, and were noticeably absent from erythrocyte progenitor populations, which were in turn populated by control or C7-treated cells (Conditions A & B). By Day 11, increased representation of all 4 treatments was observed within basophil/mast and HSC/myeloid clusters, with reduced localization within the primitive HSC fraction, implying optimal maintenance of HSCs within the first 7 days of culture.

We then quantified the effects of treatment condition on cell type by proportion (Figure 4D). Compared to DMSO controls, C7 or UM171-treated cultures demonstrated an increase in the HSC fraction which was evident on Day 3. However, an additive effect of the combination on the proportion of HSCs was observed specifically on Day 7, in line with the UMAP data. Notably, culture with UM171 (Conditions C & D) resulted in enhanced basophil/mast cell output and the depletion of erythrocyte progenitors, which were otherwise preserved in the cytokine or C7 conditions (condition A & B). Analysis of the corresponding cell proliferation data revealed increased fold expansions on Day 7 of both early HPC (CD34^BR^CD38^-^) and HSC fractions (CD90^+^ & CD49f^+^) under the combination of C7 and UM171 compared to either compound alone, although not statistically significant (Figure 4E; Figure S5). In addition, the combination resulted in increased percentages of early HPCs and HSCs relative to single treatments, particularly for CD49f^+^ HSCs (C7+UM171: 2.77% ± 0.72% vs. C7: 0.37% ± 0.16%; *P *= .005 & vs. UM171: 0.44% ± 0.24%; *P *= .006). Taken together, C7 and UM171 co-operated to expand primitive HSCs for up to 7 days *ex vivo*, although extended culture likely resulted in their differentiation toward basophil/mast cell or myeloid lineages.

Next, we focused on the 30 921 HSCs to perform UMAP again and observed that cells were primarily subclustered by the day of treatment, with Day 0 and 3 cells demonstrating distinct segregation from Days 7 and 11 (Figure S6B). To identify genes or pathways which would account for C7-mediated HSC expansion, we performed differential expression (DE) analysis using edgeR across day and condition, and filtered for genes with abs(log_2_fold-change) > 1 and FDR < 0.05 (refer to Table S4 for complete listing of DE genes). When compared to Day 0, a large number of genes (>1000) were observed to be upregulated under each treatment condition, whereas fewer genes were downregulated (Figure S4D). Conversely, when comparing C7 vs. DMSO -only conditions (B vs. A), a preponderance of DE genes (44) were observed to be down-regulated by C7 across all 3 time-points, with several almost completely suppressed (eg, *CYP1B1*, *S100A8/9/12*). In contrast, only 4 genes were upregulated to a relatively marginal degree (at most 2.2-fold) (Figure S4E).

We next performed gene set enrichment analysis (GSEA)^20^ involving pairwise comparisons between C7/DMSO (B vs. A), UM171/C7 (C vs. B), and the combination of C7 and UM171 against untreated (D vs. A) or single treatment (D vs. B, D vs. C) conditions (Table S5) using mSigDB^21^ Hallmark and curated pathway gene sets. We also included gene sets containing *CYP1B1* in our search, given its almost complete inhibition by C7 (Figure S4E) and previously reported associations with HSPC expansion.^7^^,^^27^ Among significantly (FDR < 0.05) up-regulated processes, we identified cholesterol biosynthesis and cell cycle-related pathways (Figure 4F; Figure S6A). Conversely, numerous processes relating to inflammation (TNFα/IL-10), aryl hydrocarbon (AhR) receptor or reactive oxygen species (ROS) pathways, and cellular senescence were inhibited with C7 treatment (B vs. A) (Figure 4F and G). Many of these inflammatory processes displayed increased activity under UM171 vs. C7 treatment (C vs. B), suggestive of distinct mechanisms of action. The combination of both compounds restored TNFα/NFκB and IL-10 signaling activity versus C7-only (D vs. B), while attenuating overall inflammatory responses, including STAT signaling and ROS-related metabolic processes when compared against UM171-only (D vs. C). These observations suggest a mutual regulation of inflammatory or oxidative processes by either compound, resulting in optimal levels required for enhanced expansion under the combination. Notably, TNFα and IL-10 activities were reported as decreased by C7-only treatment in our BioMAP assay (Figure S3C), consistent with the findings from GSEA.

As the molecular mechanisms of UM171 in HSPCs have been well established,^9–11^ we performed experimental validation of processes up- or down-regulated by C7. Initial investigations failed to demonstrate significant activation of the cholesterol biosynthesis pathway by C7 (Supplemental Results and Figure S6). Thus, we focused on the inflammatory and senescence-related processes down-regulated by C7. Leading edge analysis of TNFα, IL-10, and senescence pathway genes that were down-regulated for at least 1 time-point revealed several chemokines or their receptors which were either independently or jointly (eg, *CCL2*, *CXCL1* and *CXCL10*) regulated by TNFα and IL-10 signaling and inhibited by C7. A few other TNFα-regulated genes overlapped with senescence (eg, *SERPINB2*, *CDKN1A* and *F3*), suggestive of a role for NFкB-induced inflammation in inhibiting HSPC expansion (Figure 4H).

### C7 inhibits inflammation-related transcriptional changes and senescence during HSPC expansion

**Figure 5:**
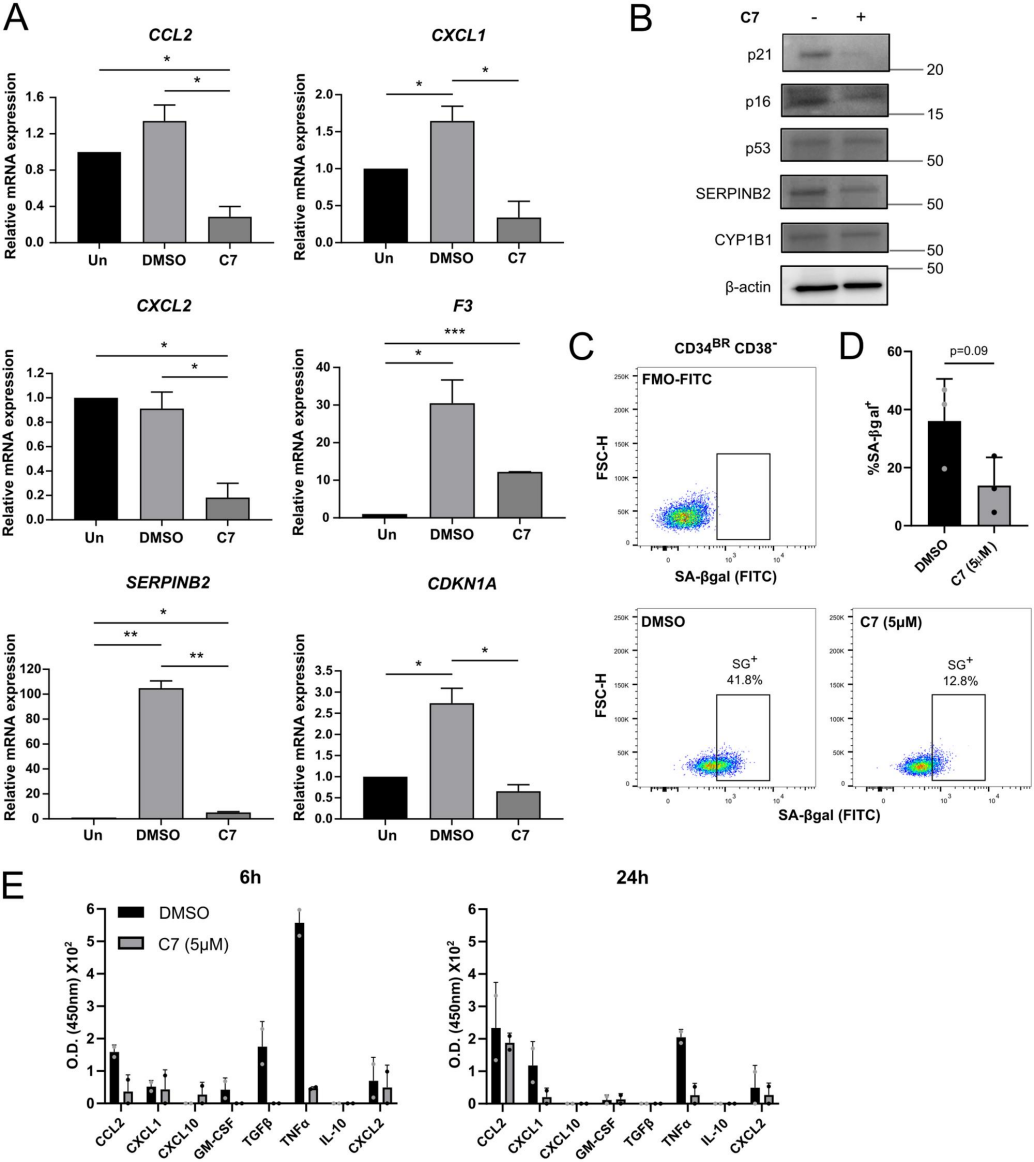
C7 inhibits molecular markers and phenotypes associated with inflammation and senescence. (A) Quantification of gene expression changes by qPCR for a single donor of UCB CD34^+^ HSPCs, unexpanded or cultured in Stemspan-AOF media with cytokines for 6h (Data are mean ± SD for *n*=4 qPCR replicates). See also Figure S7A, additional donor. (B) Western blot of cell cycle or senescence-related proteins for 1 UCB CD34^+^ sample (see also Figure S7C, 1 additional donor). (C) Representative flow cytometry plots of CellEvent Senescence Green assay for UCB CD34^BR^ CD38^-^ HSPCs cultured for 24h in Stemspan-AOF with cytokines in the presence of C7 (5µM) or DMSO for 11 days. FMO, fluorescence-minus-one control which consisted of staining for all markers excluding senescence green was used to delineate negative staining in the FITC (senescence green) channel. (D) Quantification of flow cytometry data in (C) for t=24h. Data represents mean ± SD for *N*=3 donors. See also Figure S7D for corresponding data at Days 4. 7 and 11. (E) ELISA profiling of 8 cytokines or chemokines in neat culture supernatants of UCB CD34+ HSPCs cultured in Stemspan-AOF media with cytokines and C7 (5µM) or DMSO for 6h or 24h (data are mean ± SD for *N*=2 donors). O.D., net optical density at 450nm following subtraction of background signals from media-only controls for the respective cytokines followed by mutiplication by a scaling factor of 100. See also Figure S7E for Day 4 and Day 7 data. *** indicates *P* ≤ .001,**P ≤ .01, *P ≤ .05 and n.s. (not significant) otherwise, by unpaired 2-tailed *t*-test (A) and multiple *t*-test (D) between C7 and DMSO treatments for each time-point. Abbreviations: UCB, umbilical cord blood; HSPC, hematopoietic stem and progenitor cells; AOF, Animal Origin-free; DMSO, dimethyl sulfoxide; FMO, fluorescence-minus-one; ELISA, enzyme-linked immunosorbent assay; O.D., optical density.

Next, we validated the observed transcriptional changes for selected inflammation and senescence-related genes by qPCR. DMSO-only culture of UCB CD34^+^ HSPCs resulted in early (6 hours) up-regulation of multiple genes encoding chemokines and their receptors (*CCL2*, *CXCL1,* and *CXCL2*), as well as senescence genes (*SERPINB2*, *CDKN1A,* and *F3*) relative to unexpanded (Day 0) cells. In line with the scRNA-Seq results, C7 treatment robustly and reproducibly (across 2 donors) suppressed the induction of these genes, notably *SERPINB2* (DMSO: 104.80 ± 5.82 vs. C7: 5.11 ± 0.60-fold; *P *= 1.7 × 10^−3^) (Figure 5A; Figure S7A). We also profiled longer-term gene expression changes at Days 4 to 11, demonstrating that continued C7 treatment led to prolonged suppression of these genes irrespective of any initial upregulation during DMSO-only culture (Figure S7B). Western blotting revealed that C7-mediated down-regulation of multiple senescence markers, including p16, p21, and SERPINB2, but not p53 or CYP1B1, with 24 hours of treatment (Figure 5B; S7C). In addition, flow cytometry-based analyses showed that CD34^BR^CD38^-^ early HPCs in DMSO-only cultures exhibited detectable increases in senescence-associated β-galactosidase (SA-β-gal) activity at 24 hours or following 11 days of culture, with C7 mediating more consistent reductions of SA-β-gal activity across donors at the 24 hours timepoint (24 hours DMSO: 36.07% ± 14.48% vs. C7: 13.81% ± 9.73%; *P *= .09) (Figure 5C and D; Figure S7D).

To establish if C7 could modulate the secretion of cytokines associated with a senescence-associated secretory phenotype (SASP) during *ex vivo* expansion, we performed ELISA profiling of DMSO or C7-treated UCB-CD34^+^ culture supernatants for 8 cytokines or chemokines which were (1) targets or components of NFκB or IL-10 signaling based on the GSEA data, or (2) involved in signaling pathways targeted by C7 based on our KinomeSCAN assays (eg, TGFβ), and (3) known to be associated with SASP in various contexts^28^ (Figure 5E; Figure S7E). Screening of 2 donors revealed, out of all cytokines assayed, an early increase of TNFα levels (6 hours) which declined from 24 hours onwards, and which was attenuated by C7 treatment. Taken together, our observations indicate that C7 facilitates *ex vivo* HSPC expansion by inhibiting multiple hallmarks of senescence including oxidative stress, cell-cycle arrest and inflammation. Because p21/CDKN1A can reflect altered quiescence or cell‑cycle dynamics in primitive HSPCs, we interpret decreased p21/CDKN1A in conjunction with the broader transcriptional, secretory, and enzymatic readouts (eg, SASP‑associated cytokine expression and SA‑β‑gal activity) as evidence that C7 attenuates a stress‑associated, senescence‑like program in culture rather than as proof of a single‑marker “senescence switch.”

## Discussion

The objective of this study has been to identify the mechanisms of action of our previously described azole-based small molecule C7^19^ during *ex vivo* HSPC expansion. Building on our previous study utilizing unmanipulated UCB MNCs, we observed that C7 preferentially expanded primitive subpopulations (eg, CD90^+^CD49f^+^ or CFU-GEMM) from initial CD34^+^ HSPC cultures whilst maintaining self-renewal as assessed by serial *in vivo* transplantation (Figures 1 and 2). These observations would suggest that C7 acts to restrain HSC or early HPC differentiation during *ex vivo* expansion, possibly relating to the ability of p38α to regulate cellular differentiation or stemness in a context-dependent manner.^15^ Our single-cell analyses support the ability of C7 to maintain HSCs and a balanced lineage composition during *ex vivo* expansion. In contrast, erythrocyte populations were depleted by UM171 treatment while favoring the mast cell lineage, in agreement with a recently published study^29^ and others demonstrating UM171-mediated suppression of erythroid lineage genes.^24^ Of note, during our primary transplantation assays (Figure 2D), an overall accumulation of B-cells in PB and BM over time was observed, a phenomenon previously described in the NSG mouse model.^30^ We acknowledge that the use of alternative mouse models, such as myeloid cytokine-producing/NSGS^31^ or c-Kit mutant/NSGW41^32^ may allow for more balanced or inclusive reconstitution of other lineages.

At the molecular level, our analyses identified inflammation and senescence-related pathways as being inhibited by C7 (Figure 4F). Upon inflammatory challenge, HSCs experience a loss of self-renewal and viability, as well as changes to lineage output and clonal composition.^33–36^ Senescence, a state of permanent cell cycle arrest in spite of continued metabolic activity,^28^ is another consequence of inflammation contributing to the age-related functional decline of HSCs.^37^ Our GSEA analyses underscore the notion that C7 is a potent suppressor of multiple inflammatory signaling pathways, including those involving NFкB and IL-10. Previous reports have identified rapid (within 6 hours) but transient upregulation of NFкB activity or TNFα-related gene signatures in *ex vivo* HSPC cultures.^13^^,^^38^ Genetic or pharmacological inhibition of related downstream NFкB signaling promoted HSPC expansion, self-renewal, and mitochondrial function while limiting ROS production.^13^^,^^38^^,^^39^ NFкB activation has been implicated in senescence and its associated secretory phenotype (SASP), which involves release of a host of cytokines/chemokines including IL-6, IL-8, CXCL1, and matrix metalloproteinases (MMPs) among others.^40–43^ Collectively, our data are most consistent with C7 limiting culture‑induced stress responses (oxidative/inflammatory) and attenuating senescence‑associated features during *ex vivo* expansion; additional orthogonal senescence markers would be needed to conclude definitive senescence reversal in primitive HSPC subsets.

In our study, C7 treatment of HSPCs reduced the expression of a subset of SASP cytokines, including CCL2 and CXCL1/2, which are listed as targets of NFκB and IL-10 signaling. C7 treatment mitigated an early increase in secreted TNFα protein levels during *ex vivo* culture (Figure 5E), which may reflect suppression of NFκB-induced TNFα expression by C7,^44^ so as to prevent further feed-forward effects of TNFα on proliferative arrest and senescence.^45^ Among other NFкB targets down-regulated by C7, SERPINB2 (Figure 5B) was previously identified as an intracellular protein biomarker of senescence which promotes p21 stability, cell cycle arrest and SASP in otherwise proliferating fibroblasts.^46^ Notably, the suppression of inflammation appears to distinguish C7 from other compounds with HSPC expansion capability, such as UM171 which promotes NFкB activation followed by secondary induction of EPCR to limit ROS production.^47^

Finally, while C7 efficiently inhibited p38 MAPKs, our kinase profiling work identified multiple other potential targets which may explain its superior HSPC expansion ability (Figure 3C). Among these, the TGFβ and casein kinase 1-related signaling pathways (eg, CK1α) regulate HSC self-renewal, differentiation and viability^48–52^ and are known to be responsive to stress, which may result from replication-associated DNA damage or extra-physiological oxygen exposure during *ex vivo* culture.^14^^,^^53^ A future area of investigation is to determine if and whether C7 directly inhibits TGFβRI/II or CK1δ signaling to attenuate NFкB activation and senescence in a p38-independent manner, as p38 itself may also be implicated in similar regulatory pathways.^54–56^

## Summary

Our findings suggest that C7 is a novel multi-target small molecule capable of enhancing the *ex vivo* expansion of functional human HSPCs through suppression of inflammatory and senescence-associated programs, with broad implications for regenerative medicine and cellular therapy. Furthermore, C7 could be considered as a uniformly applicable culture media supplement for stem cell expansion technologies or as an adjunct for adoptive cell therapies (CAR-T, NK cells, TILs) by improving *ex vivo* expansion and *in vivo* persistence by reducing exhaustion and SASP-like environments.

## Supplementary Material

szag037_Supplementary_Data

## Data Availability

All data related to the scRNA‑seq dataset generated in this study will be deposited in the NCBI Gene Expression Omnibus (GEO). All other data supporting the findings of this study are included in the paper and supplemental information.
