# Supplementary material for: Inhibition of inflammation and senescence with azole compound C7 enables expansion of hematopoietic stem and progenitor cells
Source: Stem Cells Transl Med. 2026 Jul 1;15(7):szag037. doi: 10.1093/stcltm/szag037 (PMC13323081; doi:10.1093/stcltm/szag037)
Supplement: szag037_Supplementary_Data [file szag037_supplementary_data.zip › Supplemental Information_20260228_LM.pdf]

Supplemental Information for:

Inhibition of inflammation and senescence with azole compound C7 enables expansion of hematopoietic stem and progenitor cells

Ze Hui Kok, Lin Ming Lee, Kelvin YH Liu, John Ouyang, Vikneswari Rajasegaran, Samantha PS Lim, Jui Wan Loh, Jing Yi Lee, Abner H Lim, Cedric Chuan Young Ng, Jason Yongsheng Chan, Sudipto Bari, William Ying Khee Hwang

Supplemental Information includes:
1. Tables S1-S3 (this file)
2. Supplemental Methods, Results and References (this file)
3. Tables S4 & S5 (.xlsx), provided as separate files

Figure S1

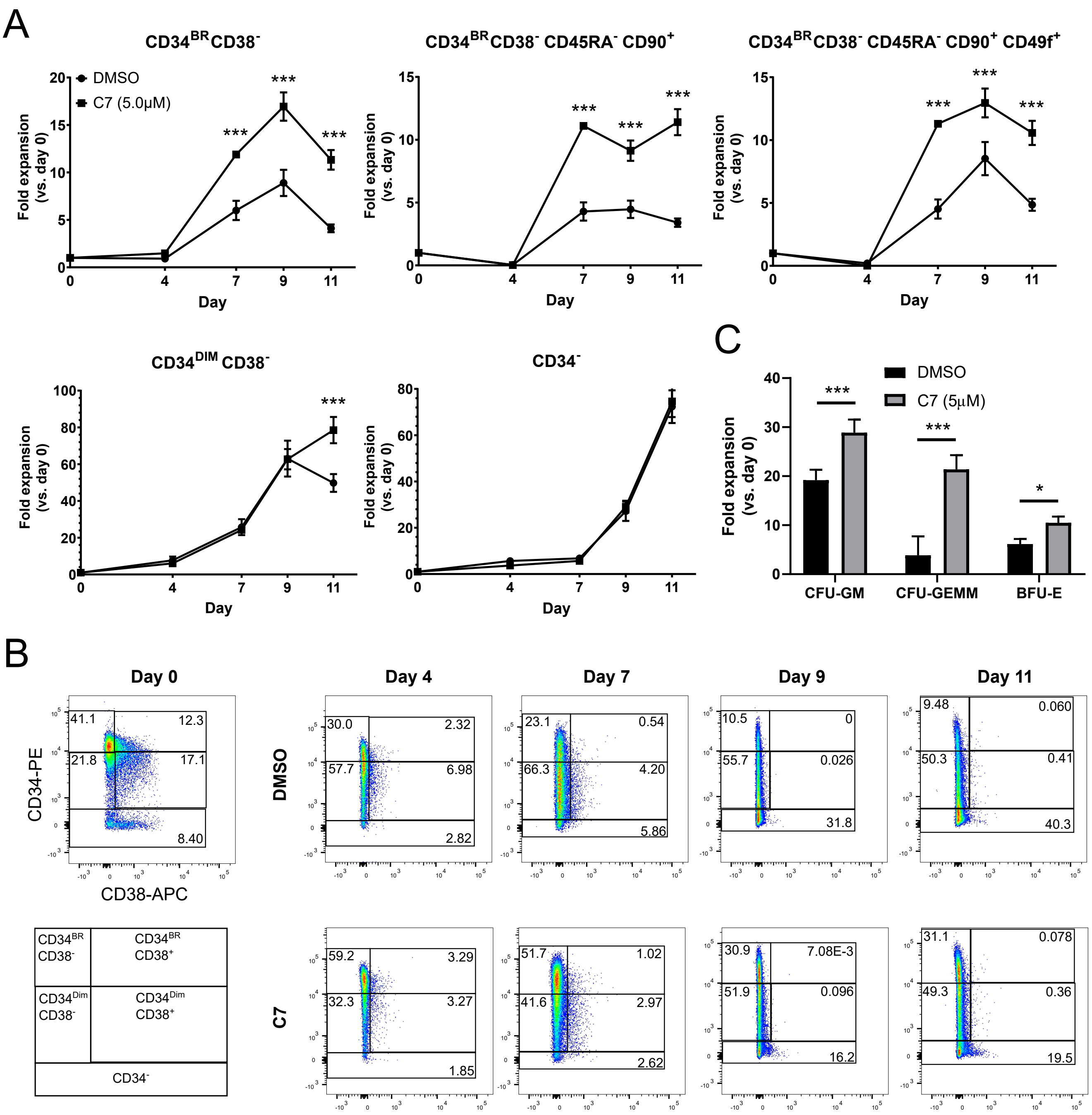

Fig. S1 Additional data demonstrating C7-mediated early HPC expansion

(A) Cell count data showing expansion of the indicated HSPC subsets (relative to Day 0) for one additional biological replicate of pooled UCB CD34^{+} HSPCs cultured with C7 (5µM) or DMSO. Data represents mean ± SD of *n*=3 counts. See also Fig. 1a.

(B) Flow cytometry data indicating C7-mediated enhancement of CD34^{BR} CD38^{-} populations.

(C) CFU assay results for one additional donor of UCB CD34^{+} HSPCs. Data represents mean ± SD of *n*=4 experimental replicates. Related to Fig. 1f.

*** indicates $p \leq 0.001$,**$p \leq 0.01$, *$p \leq 0.05$ and n.s. (not significant) otherwise, for comparisons involving C7 vs. DMSO-only controls by multiple *t*-test.

Figure S2

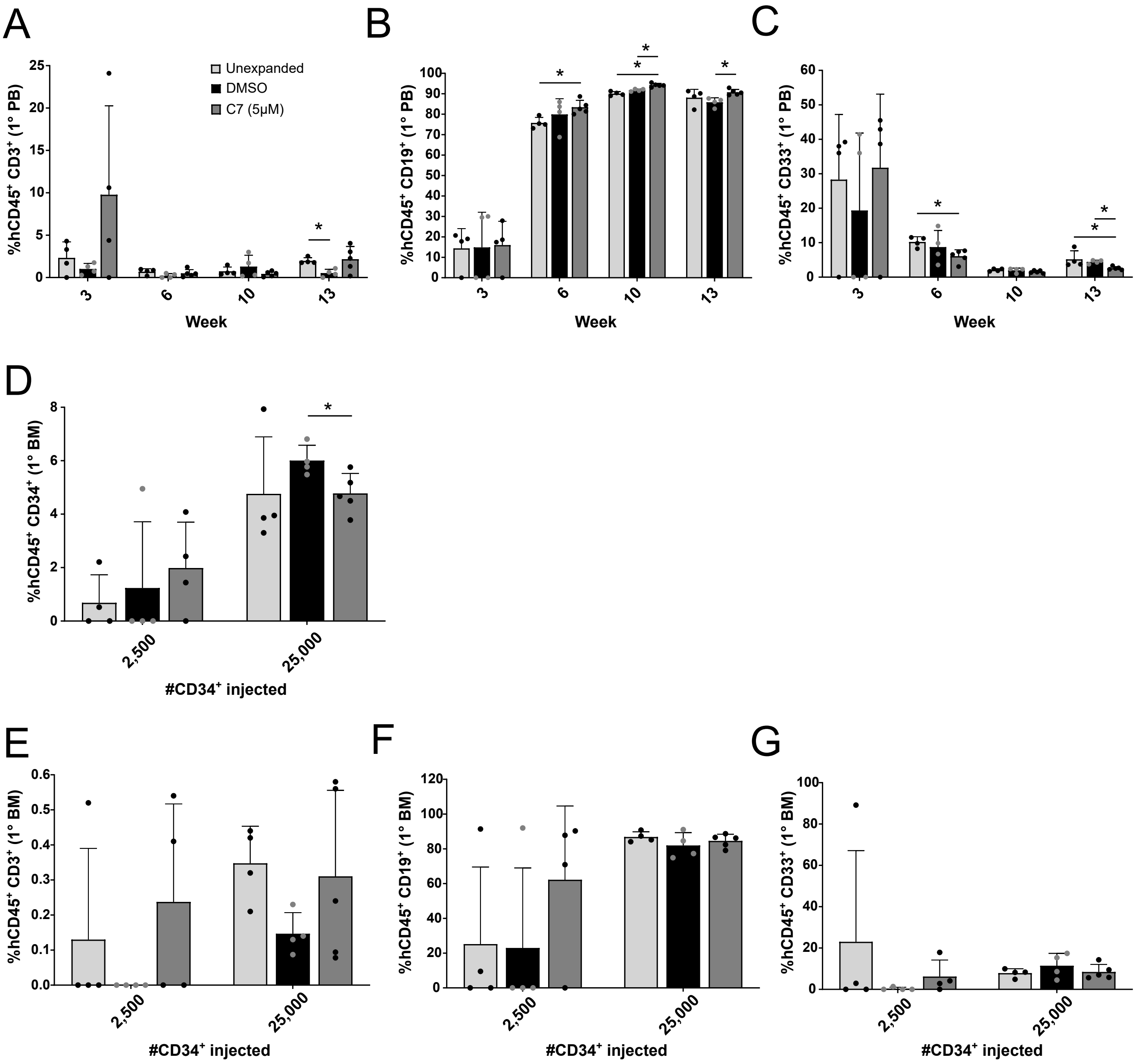

Fig. S2 Detailed primary engraftment analysis by progenitor or lineage

(A-C) Levels of T- ($CD3^+$), B- ($CD19^+$), and myeloid lineage ($CD33^+$) engraftment out of total hCD45, as indicated, in PB of primary murine recipients at Weeks 3-13. Related to Fig. 2a-b.

(D-G) Levels of progenitor ($CD34^+$), T- ($CD3^+$), B- ($CD19^+$), and myeloid lineage ($CD33^+$) engraftment out of total hCD45, as indicated, in BM of primary murine recipients at Week 16. Related to Fig. 2c.

Data represent mean ± SD for *n*=2-5 mice per treatment group. *** indicates p ≤ 0.001,**p ≤ 0.01, *p ≤ 0.05 and n.s. (not significant) otherwise, for the indicated pairwise comparisons between unexpanded, C7 and DMSO-only controls by Mann-Whitney U-test.

Figure S3

A

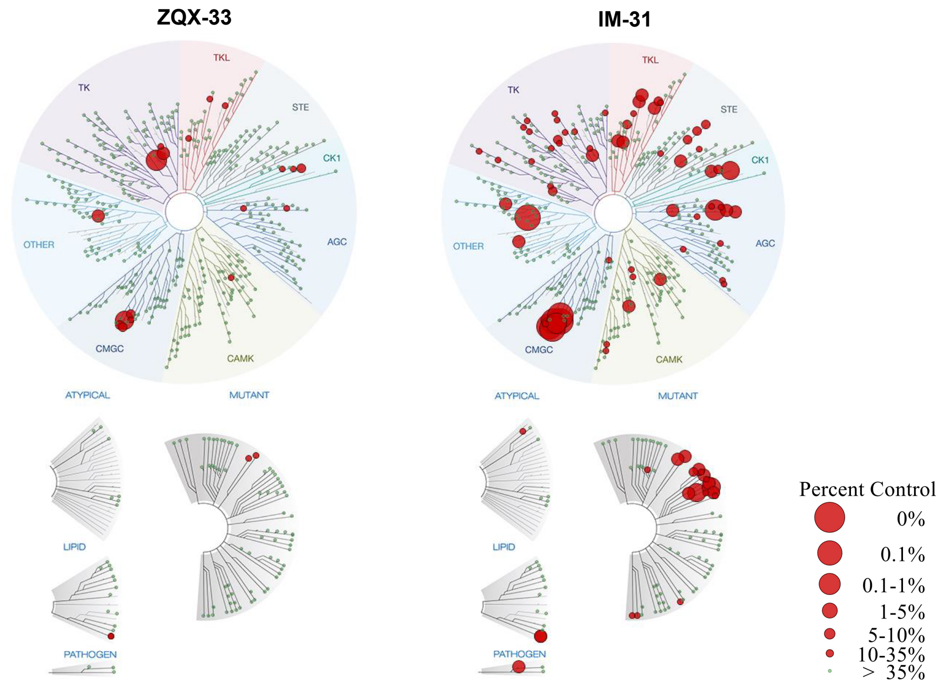

B

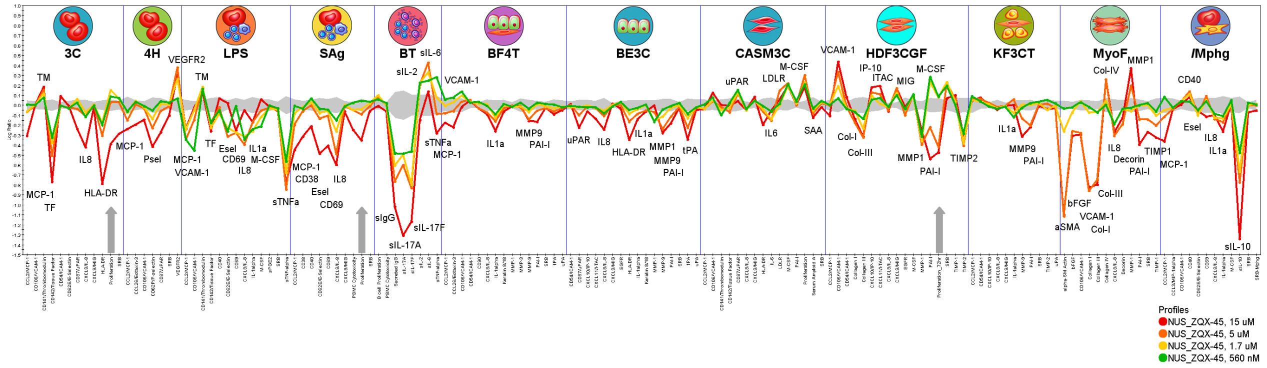

C

Biological and Disease Relevance Category	Decreased activity	Increased activity	Modulated activity
Inflammation-related activities	sTNFa, IL1a, Esel, Psel, SAA, MCP-1, IL8, IL6	IP-10, ITAC, MIG	VCAM-1
Immunomodulatory activities	HLA-DR, sIgG, sIL-10, CD38, CD69, sIL-17A, sIL-17F	sIL-6, CD40, sIL-2	M-CSF
Tissue remodeling activities	TIMP1, aSMA, TIMP2, Col-I, Col-III, tPA, MMP9, bFGF, Decorin	Col-IV	MMP1, uPAR, PAI-I
Hemostasis-related activities	TF	TM	
Other activities		VEGFR2, LDLR	

D

NUS_ZQX-45	Database Match	BioMAP Z-Standard	Pearson's Score	# of Common Readouts	Database Match (Mechanism Class)
15 µM	SB431542, 30 µM	9.728	0.668	148	TGFβR1 Kinase Inhibitor
	H-89, 10 µM	9.449	0.655	148	PKA Inhibitor
	Indacaterol Maleate, 10 µM	9.389	0.653	148	Long-acting β2 Adrenergic Receptor Agonist
5 µM	SB202190, 5.6 µM	9.332	0.707	115	p38 MAPK Inhibitor
	PD169316, 3.3 µM	8.984	0.690	115	p38 MAPK Inhibitor
	SB202190, 17 µM	8.854	0.684	115	p38 MAPK Inhibitor
1.7 µM	Doramapimod, 370 nM	11.169	0.729	148	p38 MAPK Inhibitor
	AMG548, 330 nM	11.128	0.728	148	p38 MAPK Inhibitor
	AMG548, 110 nM	10.913	0.719	148	p38 MAPK Inhibitor
560 nM	Doramapimod, 370 nM	11.370	0.737	148	p38 MAPK Inhibitor
	Doramapimod, 1.1 µM	11.101	0.727	148	p38 MAPK Inhibitor
	VX745, 1.1 µM	10.923	0.720	148	p38 MAPK Inhibitor

E

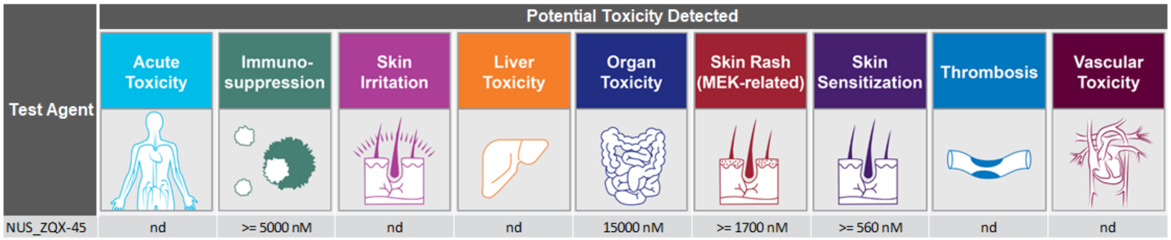

Fig. S3 Additional Treespot and BioMAP data for C7, ZQX-33 and IM-31.

(A) TreeSPOT analysis of kinase targets inhibited by control compounds ZQX-33 and IM-31.

(B) BioMAP profile of C7 (NUS_ZQX-45) in the Diversity PLUS Panel. The X-axis indicates quantitative protein-based biomarker readouts measured in each system. The Y-axis indicates the log-transformed ratio of biomarker readouts for C7-treated cells (n = 1) vs. vehicle controls ($n \geq 6$). The gray region delineates a 95% significance interval defined using previous vehicle controls. Thick gray arrows indicate antiproliferative effects.

(C) C7-mediated changes in key biomarker activities, listed by biological and disease classifications.

(D) The top matches from BioMAP Reference Database demonstrating that C7 exhibits mechanistic similarity to p38-MAPK inhibitors at concentrations of 5µM and below. For each pair of compound profiles, similarity was determined by filtering and ranking (BioMAP Z-Standard) the Pearson's correlation coefficient. A pair is considered mechanistically similar if the Pearson's correlation coefficient is ≥ 0.7.

(E) Toxicity Signatures detected within C7 BioMAP profiles at the concentrations tested. nd, not detected; na, not assessed (e.g. due to excessive cytotoxic behavior).

Figure S4

A

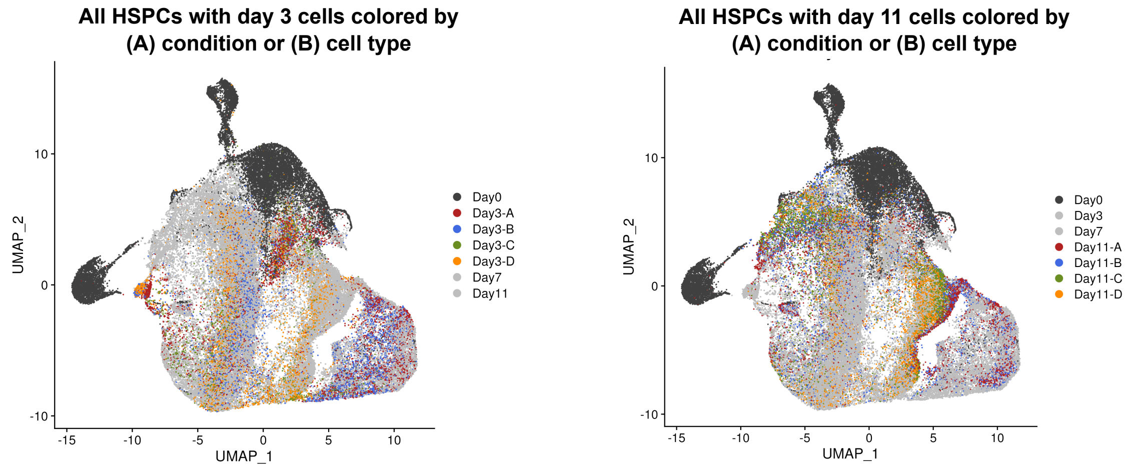

B

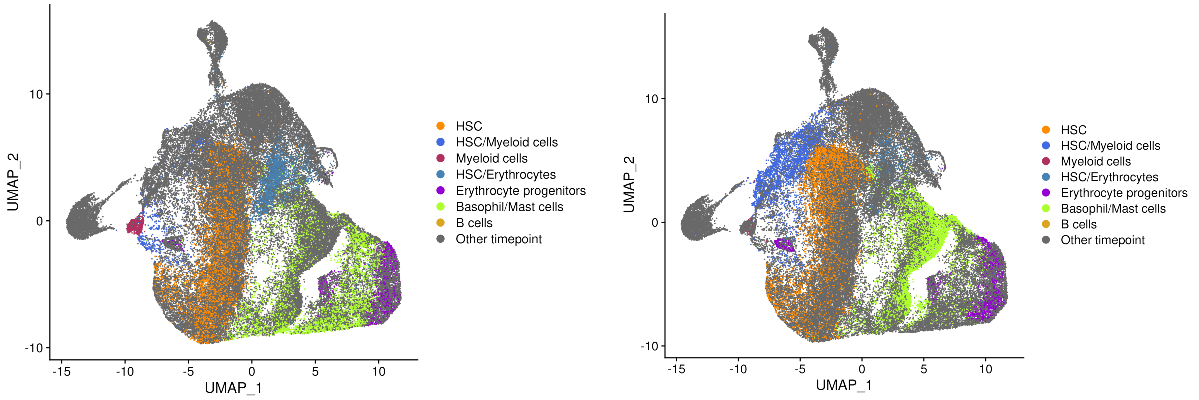

C

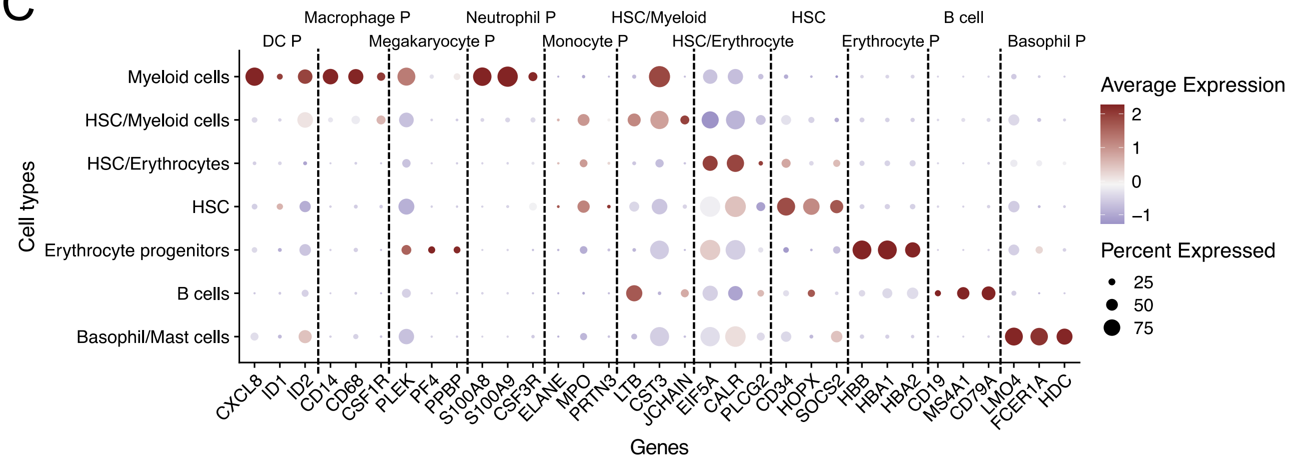

D

Differential expression across day

Number of genes that passed FDR < 0.05

	Up-regulated (logFC > 1)				Down-regulated (logFC < -1)			
	A	B	C	D	A	B	C	D
Day0-3	1540	1136	1808	1062	417	520	474	424
Day0-7	1221	1093	1276	1408	409	425	251	235
Day0-11	1243	1102	988	1442	160	140	113	115
Day0 (shared)	672	507	612	712	93	92	82	67
Day3-7	62	117	124	148	156	110	189	140
Day3-11	313	524	326	271	133	75	164	42
Day3 (shared)	39	103	97	107	48	14	67	25
Day7-11	240	338	128	148	18	37	22	20

E

Log2 (Fold change) C7 vs. DMSO

-7.94 — 0 — 1.14

Group	Day3	Day7	Day11	Gene
C7 DOWN (44)	-6.35	-7.05	-6.57	CYP1B1
	-7.24	-7.94	-4.06	S100A8
	-6.69	-5.08	-4.41	S100A12
	-4.99	-5.44	-4.80	S100A9
	-2.64	-4.53	-3.94	CD14
	-7.04	-2.51	-2.73	CLEC10A
	-3.54	-2.58	-2.14	LYZ
	-3.83	-2.21	-2.17	CLEC5A
	-4.24	-1.64	-2.20	CCR2
	-4.03	-1.66	-1.52	ASGR2
	-1.81	-2.68	-1.73	MMP2
	-2.94	-1.94	-1.46	RNASE3
	-3.42	-1.72	-1.40	ALOX5AP
	-2.03	-1.82	-1.94	PRDM1
	-2.92	-1.18	-2.21	HBG2
	-1.85	-2.57	-1.49	MNDA
	-2.71	-1.70	-1.37	RNASE2
	-1.16	-2.37	-2.07	EPB41L3
	-2.04	-1.38	-1.75	IL10RA
	-2.53	-1.24	-1.53	HNMT
	-1.91	-1.85	-1.28	AOAH
	-3.02	-1.66	-0.88	CSTA
	-2.26	-1.23	-1.31	ABI3
	-3.64	-1.11	-1.00	SKIL
	-1.96	-1.52	-0.79	AHRR
	-1.32	-1.50	-1.18	B3GNT7
	-2.31	-1.36	-0.72	S100B
	-1.90	-1.25	-0.91	GPR183
	-1.66	-1.01	-0.99	RASGRP3
	-1.63	-1.10	-0.88	SEMA4A
	-1.05	-1.57	-0.94	ANPEP
	-1.57	-0.85	-1.01	TYMP
	-1.55	-1.21	-0.64	RASSF4
	-1.04	-0.94	-1.29	LACC1
	-1.53	-0.83	-0.85	AC080038.1
	-0.70	-0.98	-1.39	SLC2A5
	-0.80	-0.97	-0.77	QSOX1
	-0.99	-1.05	-0.52	CEBPD
	-1.09	-0.75	-0.66	PRKCD
	-0.93	-0.77	-0.66	FLNA
	-0.93	-0.74	-0.62	EMILIN2
	-0.86	-0.83	-0.58	MAN2B1
	-0.68	-0.61	-0.58	ID2
	-0.65	-0.58	-0.53	CEBPA
C7 UP (4)	0.58	0.54	0.64	CD52
	0.61	0.64	0.72	TSTD1
	1.14	0.79	0.89	HEMGN
	1.01	0.98	1.08	IFITM1

Fig. S4 Supporting information for scRNA-seq and gene expression analysis

(A) UMAP plots of all HSPCs colored by treatment (A-D) for Day 3 (left) or Day 11 (right). HSPCs from Day 0 or other timepoints are colored in black and grey, respectively. Related to Fig. 4b.

(B) UMAP plots of Day 3 (left) or Day 11 (right) HSPCs colored by cell type, with clustering based on marker genes as defined in Fig. S4c. HSPCs from other timepoints are colored in grey. Related to Fig. 4c.

(C) List of features consisting of previously reported[1,2] marker genes detected in HSPCs, used to define cell type clusters in Fig. 4c. Genes are colored according to average expression among cells in each cluster, and sized according to the percentage of cells from each cluster expressing the gene.

(D) Summary of the number of genes up- (logFC >1) or down-(logFC<-1) regulated by treatments A-D, relative to Day 0 (yellow), Day 3 (blue) or Day 7 (green). For each treatment, “shared” genes indicate genes up- or down-regulated at multiple timepoints relative to Day 0, 3 or 7 as indicated.

(E) Heatmap of genes up- (Log_2 FC > 0) or down- (Log_2FC < 0) regulated by C7 treatment relative to DMSO controls in the HSC fraction. Only genes up- (44) or down- (4) regulated at all 3 time-points are listed. The Log_2FC for B vs. A conditions for each gene is indicated by the number in the respective box. Genes have been ranked according to their average Log_2-fold changes across all 3 time-points. *CYP1B1* and *IFITM1* are the most strongly up- and down-regulated genes, respectively.

Figure S5

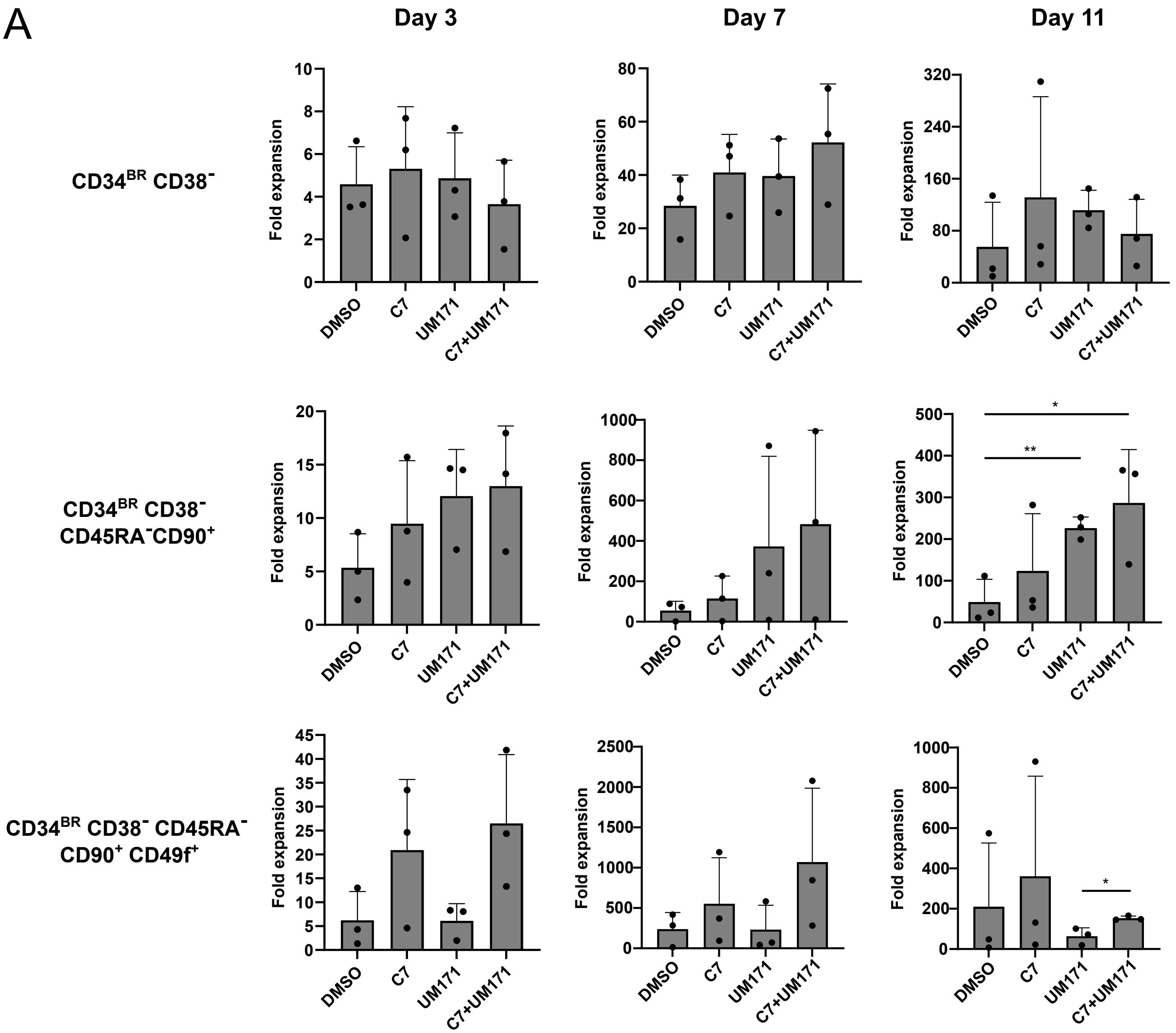
A
Day 3
Day 7
Day 11
CD34^{BR} CD38^{-}
CD34^{BR} CD38^{-} CD45RA^{-}CD90^{+}
CD34^{BR} CD38^{-} CD45RA^{-} CD90^{+} CD49f^{+}
Fold expansion
DMSO
C7
UM171
C7+UM171
*
**

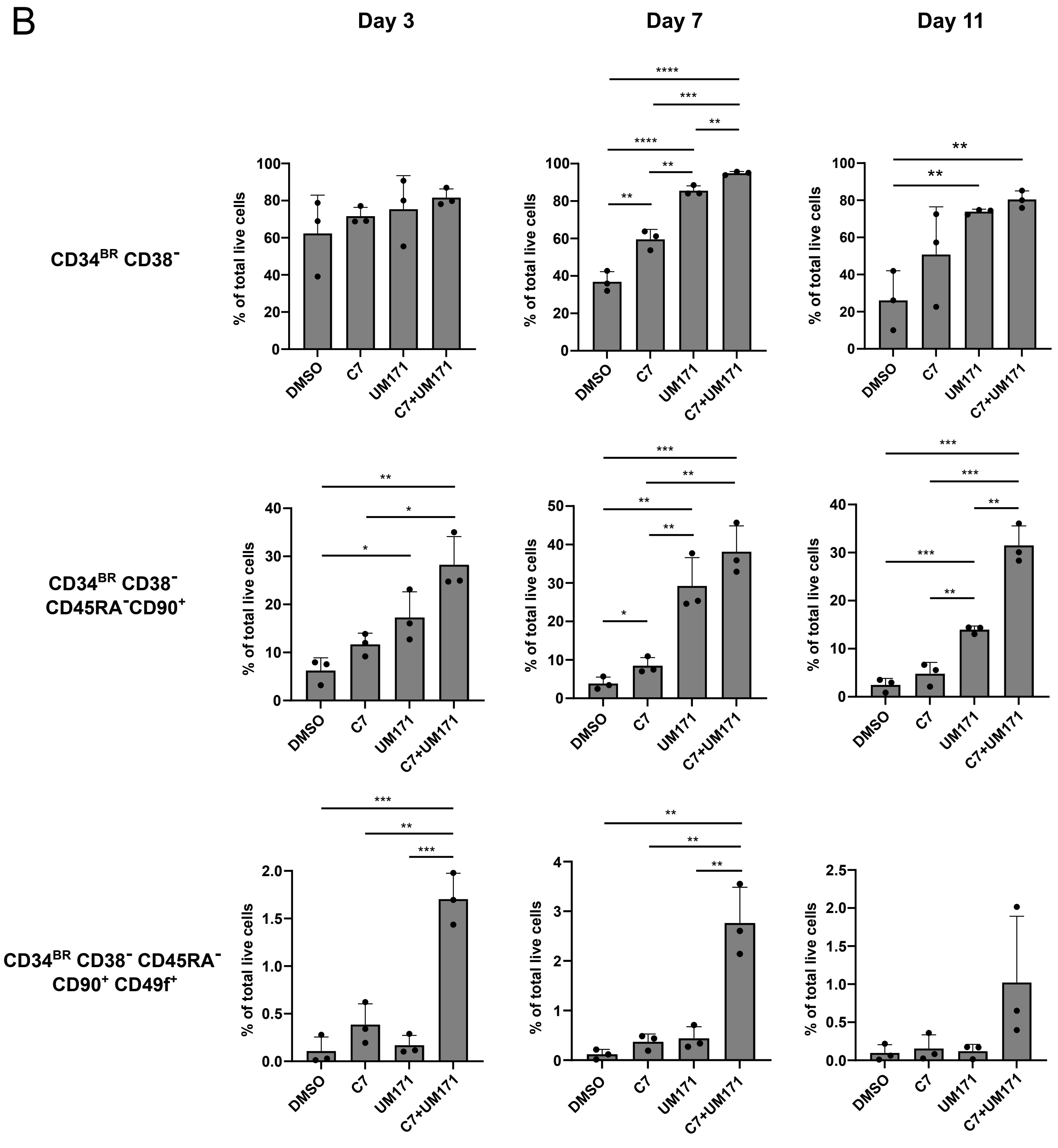

Fig. S5 C7 and UM171 co-operate to enhance HSC maintenance during *ex vivo* expansion

(A) Fold expansion of early HPCs ($CD34^{BR}$ $CD38^{-}$) and HSCs ($CD34^{BR}$ $CD38^{-}$ $CD45RA^{-}$ $CD90^{+}$ ± $CD49f^{+}$) fractions at Days 3, 7 or 11, in response to C7 (5μM) or UM171 (50nM) treatments as well as the combination of both compounds. Calculations are made relative to Day 0 starting cultures of UCB-$CD34^{+}$ HSPCs. Data represent mean ± SD for *N*=3 donors. Related to Fig. 4e.

(B) % of the early HPC or HSC fractions as described in (A), out of total live cells ($7\text{-}AAD^{-}$) as determined by flow cytometry.

*** indicates p ≤ 0.001,**p ≤ 0.01, *p ≤ 0.05 and n.s. (not significant) otherwise, for comparisons involving C7 vs. DMSO-only controls by unpaired two-tailed *t*-test.

Figure S6

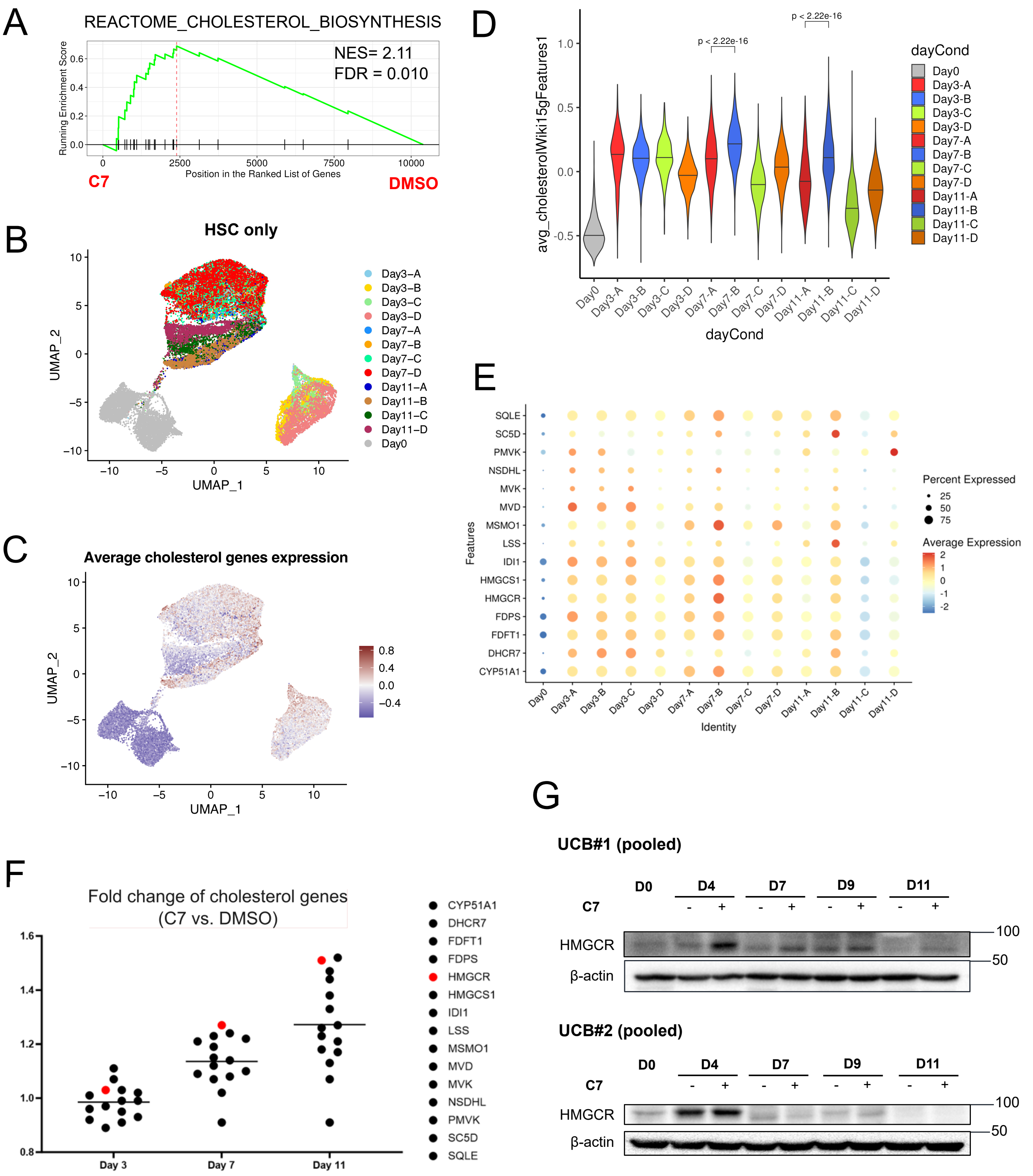
A
REACTOME_CHOLESTEROL_BIOSYNTHESIS
NES= 2.11
FDR = 0.010
Running Enrichment Score
Position in the Ranked List of Genes
C7
DMSO
B
HSC only
UMAP_1
UMAP_2
C
Average cholesterol genes expression
D
avg_cholesterolWiki15gFeatures1
p < 2.22e-16
p < 2.22e-16
dayCond
E
Percent Expressed
Average Expression
Features
Identity
SQLE
SC5D
PMVK
NSDHL
MVK
MVD
MSMO1
LSS
IDI1
HMGCS1
HMGCR
FDPS
FDFT1
DHCR7
CYP51A1
F
Fold change of cholesterol genes
(C7 vs. DMSO)
Day 3
Day 7
Day 11
G
UCB#1 (pooled)
UCB#2 (pooled)
D0
D4
D7
D9
D11
C7
HMGCR
β-actin
100
50

H

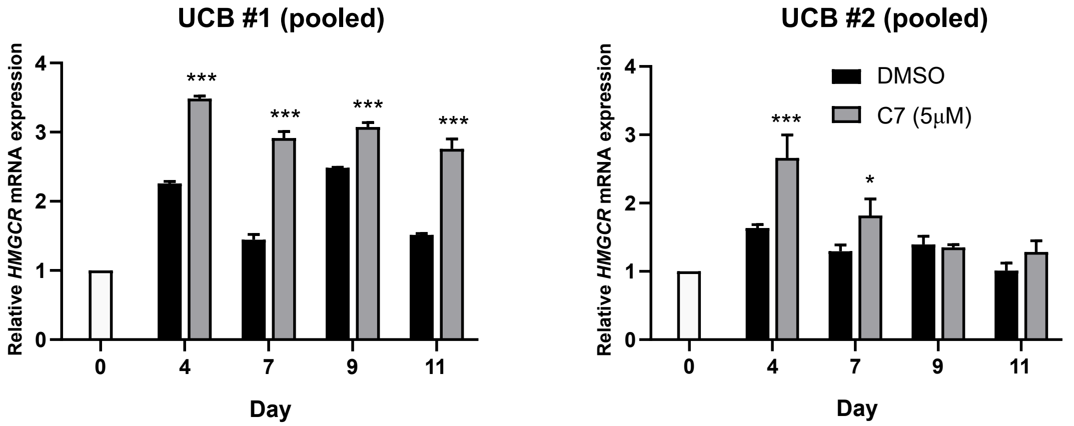
UCB #1 (pooled)
UCB #2 (pooled)
Relative *HMGCR* mRNA expression
DMSO
C7 (5μM)
***
*
0
4
7
9
11
Day

I

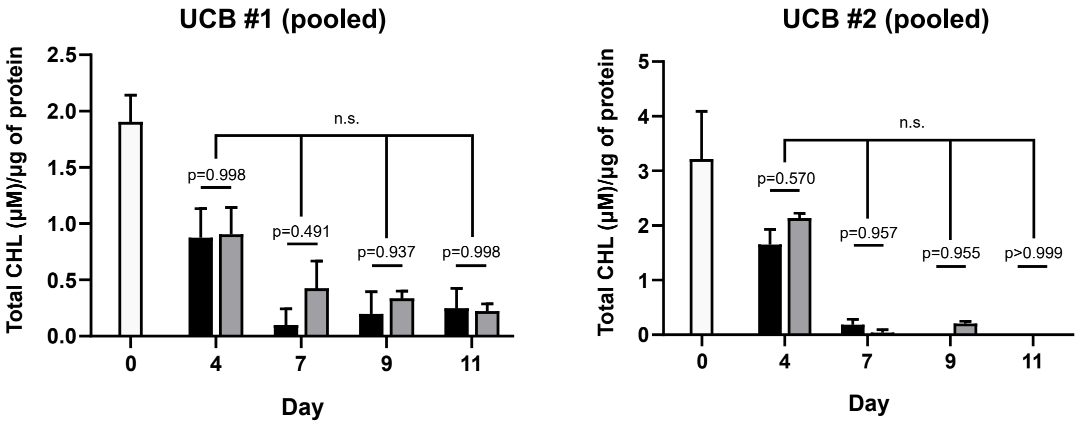
UCB #1 (pooled)
UCB #2 (pooled)
Total CHL (μM)/μg of protein
n.s.
p=0.998
p=0.491
p=0.937
p=0.998
p=0.570
p=0.957
p=0.955
p>0.999
0
4
7
9
11
Day

Fig. S6 Supporting information for investigations on C7-mediated enrichment of cholesterol biosynthesis

(A) GSEA plot demonstrating positive enrichment of cholesterol biosynthesis pathway in C7 relative to DMSO-only condition.

(B) UMAP plot of HSCs colored by day and treatment group. Day 0 HSCs are colored in grey.

(C) UMAP plot of HSCs colored by average expression of 15 cholesterol pathway genes as defined by WikiPathways (see Results section).

(D) Violin plot depicting average cholesterol gene expression as in (C), according to treatment condition and time-point. *p*-values were generated by Wilcoxon signed-rank test.

(E) Bubble enrichment plot showing, based on color, the average expression of individual cholesterol pathway genes in HSCs grouped by day and treatment. The percentage of HSCs expressing each gene is indicated by the node size.

(F) Scatter plot depicting fold change of individual cholesterol genes (C7 vs. DMSO) for each time-point. Horizontal lines indicate average expression values for all genes. *HMGCR* expression (above average) is highlighted in red.

(G) Western blot of HMGCR protein expression from 2 biological replicates in response to treatment with C7 (5μM) or DMSO for the indicated durations. Each replicate consists of pooled $CD34^+$ HSPCs from multiple donors.

(H) qPCR analysis of HMGCR gene expression changes in response to C7 or DMSO-only treatment, normalized to Day 0. Data are mean ± SD from 2 independent pooled biological replicates as in (G), with 2 technical repeats each (total n=4).

(I) Assay for total cholesterol levels in $CD34^+$ HSPCs from 2 biological replicates with 2 technical repeats each (total n=4), in response to DMSO or C7 treatment.

*** indicates p ≤ 0.001,**p ≤ 0.01, *p ≤ 0.05 and n.s. (not significant) otherwise, for comparisons involving C7 vs. DMSO-only controls by multiple *t*-test.

Figure S7

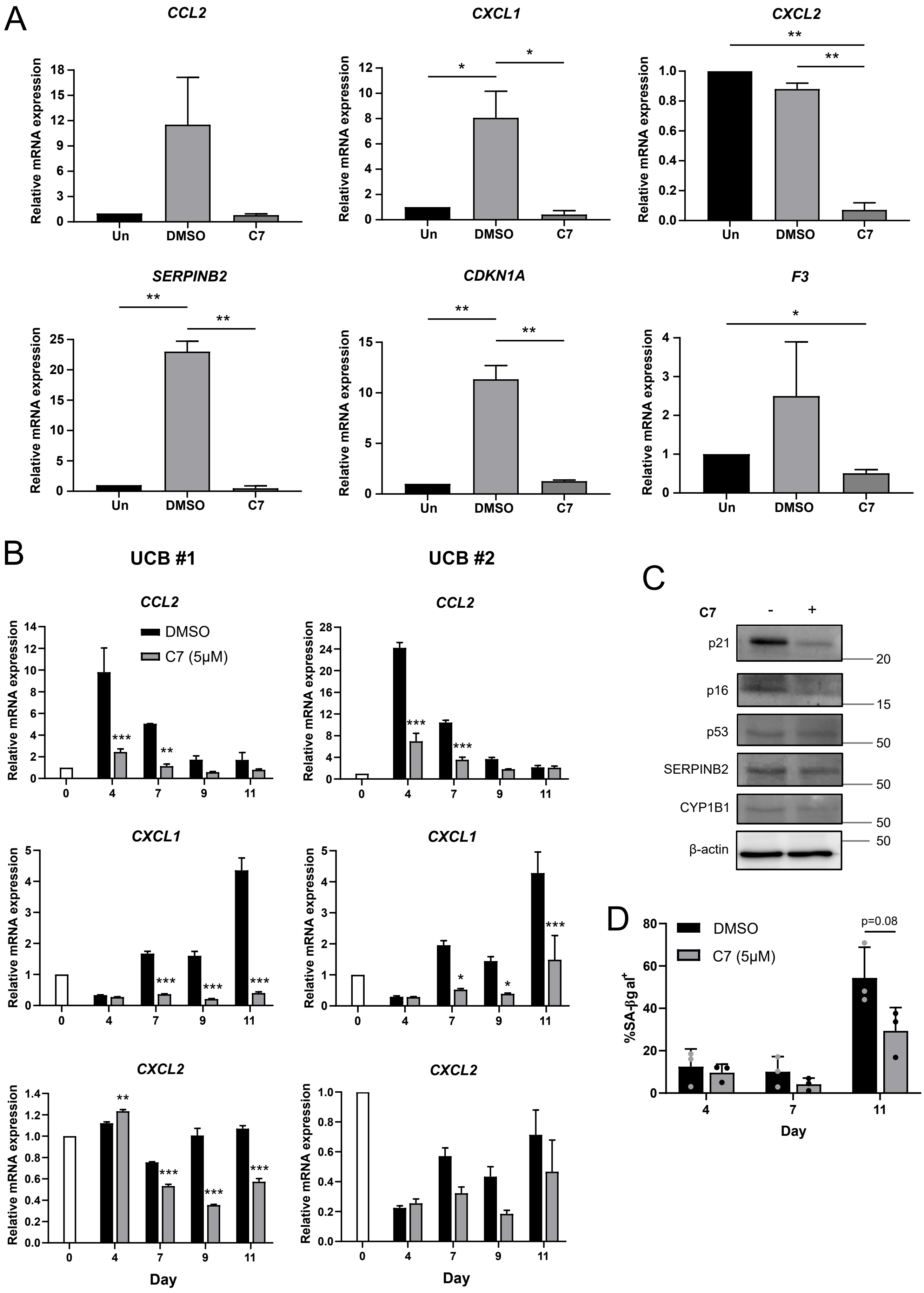
A
CCL2
CXCL1
CXCL2
SERPINB2
CDKN1A
F3
Relative mRNA expression
Un
DMSO
C7
B
UCB #1
UCB #2
CCL2
CXCL1
CXCL2
DMSO
C7 (5μM)
Day
C
C7
-
+
p21
p16
p53
SERPINB2
CYP1B1
β-actin
20
15
50
D
%SA-βgal+
p=0.08
4
7
11

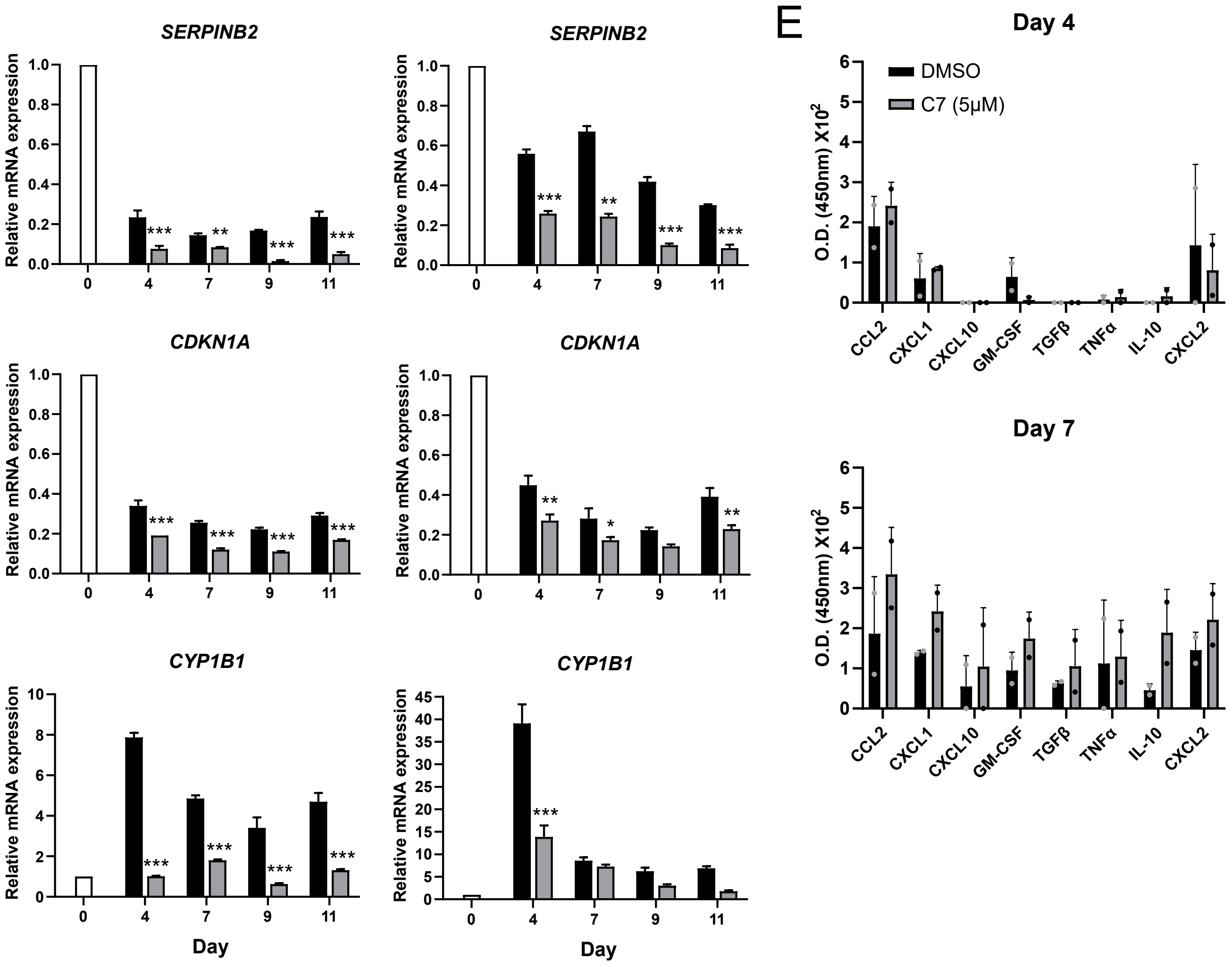

Fig. S7 Extended validation of inflammation and senescence gene and protein expression

(A) Quantification of expression changes for the indicated genes (t= 6h) (as in Fig. 5a) for one additional donor of UCB CD34$^+$ HSPCs (mean ± SD of *n*=4 qPCR replicates). Un, untreated HSPCs.

(B) Quantification of expression changes for the indicated genes (Day 4 to 11) for two pooled donor replicates of UCB CD34$^+$ HSPCs (mean ± SD of *n*=4 qPCR replicates per pool).

(C) Western blot of cell cycle or senescence-related proteins for one additional UCB CD34$^+$ sample (related to Fig. 5b).

(D) Quantification of SA-βgal activity by flow cytometry (CellEvent Senescence Green) in UCB CD34^{BR} CD38$^-$ HSPCs cultured with C7 or DMSO for 4, 7 or 11 days (mean ± SD for *N*=3 donors). Related to Fig. 5d.

(E) ELISA profiling of 8 cytokines or chemokines in culture supernatants of UCB CD34$^+$ HSPCs treated with DMSO or C7 for 4 or 7 days (*N*=2 donor replicates). O.D., net optical density at 450nm following subtraction of background signals from media-only controls for the respective cytokines followed by mutiplication by a scaling factor of 100. Related to Fig. 5e.

*** indicates p ≤ 0.001,**p ≤ 0.01, *p ≤ 0.05 and n.s. (not significant) otherwise, by (A) unpaired two-tailed *t*-test; (B) and (D) multiple *t*-test between C7 and DMSO-only treatments for each time-point.

Table S1: List of reagents used for flow cytometry and Western blotting

(a) Flow cytometry: UCB-HSPCs

Antibody or reagent	Source	Catalog no.
7-AAD	Beckman Coulter, USA	IM3422
hCD45-PE-Cy7	BD Biosciences, USA	557748
hCD34-PE	Miltenyi Biotec, Germany	130-113-179
hCD38-APC	BD Biosciences, USA	555462
hCD45RA-V450	BD Biosciences, USA	560362
hCD90-FITC	BD Biosciences, USA	555595
hCD49f-PerCP-Cy5.5	BD Biosciences, USA	562475

(b) Flow cytometry: Animal experiments

Antibody or reagent	Source	Catalog no.
hCD45-APC	Miltenyi Biotec, Germany	130-113-114
mCD45-Viogreen	Miltenyi Biotec, Germany	130-123-900
hCD3-FITC	Miltenyi Biotec, Germany	130-113-128
hCD19-PE-Vio615	Miltenyi Biotec, Germany	130-114-521
hCD33-PE-Vio770	Miltenyi Biotec, Germany	130-113-350
hCD7-APC-Cy7	Miltenyi Biotec, Germany	130-117-677
hCD34-PE	Miltenyi Biotec, Germany	130-113-179

(c) Western Blotting

Antibody or reagent	Source	Catalog no.
Rabbit α-human p-p38 (T180/Y182)	Abcam, UK	ab4822
Rabbit α-human total p38	Cell Signaling Technology, USA	8690
Mouse α-human HMGCR	Abcam, UK	ab242315
Rabbit α-human p21	Cell Signaling Technology, USA	2947
Mouse α-human CYP1B1	Santa Cruz Biotechnology, USA	sc374228
Mouse α-human SERPINB2	Santa Cruz Biotechnology, USA	sc166539
Mouse α-human β-actin	Cell Signaling Technology, USA	3700
α-Rabbit IgG HRP	Cytiva, USA	NA934-1ML
α-Mouse IgG HRP	Cytiva, USA	NA931-1ML

Table S2: Numbers of UCB $CD34^+$ HSPCs used for primary transplantations

	No.(#) of $CD34^+$ HSPCs injected/mice (*N*) in each group											
Treatment group	Day 0 unexpanded				Day 11 expanded (DMSO)				Day 11 expanded (C7 - 5μM)			
	TNC	%$CD34^+$	#$CD34^+$	*N*	TNC ExF: 21	%$CD34^+$	#$CD34^+$	*N*	TNC ExF: 25	%$CD34^+$	#$CD34^+$	*N*
250	250	94.2%	233	4	5250	83.4%	4365	4	6250	87.6%	5459	4
2500	2500		2327	4	52500		43654	4	62500		54586	4
25000	25000		23267	4	525000		436536	4	625000		545858	5

TNC, Total nucleated cells

Table S3: List of qRT-PCR primers used

**Gene** (5'→3')	**Primer sequence**
GAPDH_F	AACAGCGACACCCACTCCTC
GAPDH_R	CATACCAGGAAATGAGCTTGACAA
ACTB_F	CTGGAACGGTGAAGGTGACA
ACTB_R	GTCCTCGGCCACATTGTGAA
MAPK14_F (p38)	TAACAGGATGCCAAGCCATGAG
MAPK14_R (p38)	GCTTGGGCCGCTGTAATTC
HMGCR_F	TGTTCATGCTCACAGTCGCT
HMGCR_R	GCCAGAGGGAAACACTTGGT
CCL2_F	GATCTCAGTGCAGAGGCTCG
CCL2_R	TTTGCTTGTCCAGGTGGTCC
CXCL1_F	AGGCAGGGGAATGTATGTGC
CXCL1_R	GCCCCTTTGTTCTAAGCCAG
CXCL2_F	GAAAGCTTGTCTCAACCCCG
CXCL2_R	TGGTCAGTTGGATTTGCCATTTT
SERPINB2_F	TGCTTCCAGATGAAATTGCCG
SERPINB2_R	TCCATGCCCATGCTTCTCAG
F3_F	CGGAAGAGTACAGACAGCCC
F3_R	CTCCAGGGTCTTCATGCTCC
CDKN1A_F	GGCTGGGAGTAGTTGTCTTTC
CDKN1A_R	TCGAGAGGTTTACAGTCTAGGT

Supplemental Methods

KINOMEscan and BioMAP Diversity PLUS Profiling

Lyophilized C7 was reconstituted in sterile DMSO (Sigma Aldrich, USA; D2438) to 20 mM and transported at room temperature to Eurofins DiscoverX (USA) for KINOMEscan assay and BioMAP Diversity PLUS panel with Toxicity Signature Analysis.

For KINOMEscan, C7 was diluted in DMSO to 5μM and its binding affinity was tested against a panel of 468 kinases. Results were then compared with two other structural analogs of C7, ZQX-33 and IM-31 which were prepared similarly. The extent of kinase-ligand binding inhibition was presented as percent of control (%Ctrl), according to the equation: % Ctrl = [(Test compound signal - Positive control signal) / (Negative control signal - Positive control signal)] × 100, where positive control is the control compound (0% Ctrl) and negative control is DMSO (100% Ctrl), and lower %Ctrl indicates stronger hits.

To test against the BioMAP Diversity PLUS panel, C7 (ZQX-45) was further diluted into testing concentrations of 15 μM, 5 μM, 1.7 μM, 560 nM. The Diversity PLUS panel is a commercially validated service encompassing 12 distinct systems that consist of diverse cell types and their adaptive responses to inflammatory, proliferative and infectious stimuli. These systems allow testing of drugs and compounds for their effects on clinically relevant protein markers and model the disease states that result from pharmacological interventions. Activity of the C7 compound was mathematically compared against the BioMAP Reference Database of > 4,500 agents in an unsupervised search for the top three compounds that matched similarly to C7. Against C7 testing concentrations of 15 μM, 5 μM, 1.7 μM and 560 nM, the top database matching profiles were determined using a combinatorial approach which involves filtering (Tanimoto metric) and ranking (BioMAP Z-Standard) the Pearson’s correlation coefficient between two profiles. Profiles were identified as having mechanistically relevant similarity if the Pearson’s correlation coefficient was

≥ 0.7. BioMAP Toxicity Signature Analysis was also conducted on C7 to observe toxicity and pharmacology signatures on various systems.

Cholesterol assay

UCB-CD34$^+$ cell pellets of 4 x 10^5 each were stored at -80°C for subsequent cholesterol quantitation via the Cholesterol/Cholesterol Ester-Glo assay (Promega, USA; J3191). 50µl of Cholesterol Lysis Solution was added to each cell pellet followed by an incubation of 30 mins at 37°C. Next, 10µl per sample was added to each well of a 96-well white clear bottom plate in duplicate. 10µl of cholesterol detection reagent with esterase was added per well, followed by shaking for 1 min before incubation at room temperature for 1 hour. Thereafter, luminescence readings were recorded using a Tecan Infinite 200 PRO (Tecan, Switzerland) plate reader at 2200ms intervals.

scRNA-seq sample preparation, sequencing, data processing and analysis

UCB-CD34$^+$ cells were cultured with C7 or DMSO as described previously for 3, 7 and 11 days. Harvested cells were then resuspended into single-cell suspensions into 1000-6000 cells/µl to be loaded into Chromium microfluidic chips with targeted cell recovery of 10,000 using the Chromium Controller (10x Genomics, USA). Single-cell RNA-seq libraries were prepared according to the manufacturer's instructions using the Chromium Next GEM Single Cell 3' Reagent Kits v3.1 (Dual Index) (10 X Genomics, USA; PN-1000268). Captured libraries were sequenced on the Novaseq 6000 sequencer analyzer at PE150 and sequencing depth of 20,000 read pairs per cell (Novogene, China).

The demultiplexed reads were aligned against GRCh38 reference genome using Alevin via salmon 1.5.2 [1,2] . The single-cell data were loaded into count matrices, and all 45 samples were merged using Seurat v4.1 [3]. Cells that expressed 500 to 6,702 (top 2% of entire dataset) genes and contained less than 20% of transcripts mapping to mitochondrial genes were retained. Subsequently, 5,000 cells were subsampled from each day and condition (13 day-conditions in

total), with the exception of Day3-C, which is comprised of 2,151 cells, and Day0, where we subsampled 15,000 cells. This resulted in a total of 72,151 cells, which were then subjected to downstream preprocessing including scaling, log-normalization of gene expression measurements, and selection of the top 2,000 highly variable genes for principal component analysis (PCA) as implemented by Seurat. UMAP was used to visualize the distribution of cells using the top PCs determined from an elbow plot, and cell type annotation was performed based on the marker genes detected in hematopoietic stem and progenitor cells from Lai *et al*. [4,5].

Among HSCs, differential expression analysis, using edgeR [6–8], was performed across day and condition with filtering criteria of FDR < 0.05 and abs(log2-fold-change) > 1, and the up or down-regulated genes were used for gene enrichment analysis (see Methods).

Supplemental Results

C7 induces minimal activation of the cholesterol biosynthesis pathway in UCB-HSPCs

The cholesterol biosynthesis pathway is known to involve the enzyme HMG-CoA reductase, commonly targeted by statins [9]. We analyzed the expression of 15 genes from the WP_CHOLESTEROL_BIOSYNTHESIS_PATHWAY of WikiPathways (*CYP51A1, DHCR7, FDFT1, FDPS, HMGCR, HMGCS1, IDI1, LSS, MSMO1, MVD, MVK, NSDHL, PMVK, SC5D*, and *SQLE*) and calculated the cholesterol biosynthesis pathway activity using the AddModuleScore function in Seurat. UMAP analysis revealed an increase in the average expression of cholesterol pathway genes from Day 3 compared to untreated (Day 0) cells (Fig. S6c). These differences were statistically significant ($p < 0.001$) when comparing Day 7 or 11 C7 vs. DMSO-only treatments, whereas no increases were observed with the addition of UM171 (Fig. S6d). At the individual gene level, three (*HMGCR*, *HMGCS1,* and *MSMO1*) were found to exhibit increased expression in a majority of C7-treated (Day 7) cells (Fig. S6e). Among these, *HMGCR* was the most highly upregulated gene in response to C7 treatment at Day 7 (1.45-fold) or 11 (1.76-fold),

compared to DMSO-only controls (Fig. S6f). However, we did not observe consistent or significant increases in *HMGCR* mRNA or protein based on qPCR or Western blotting (Fig. S6g-h), and C7 did not mediate any increases in total cholesterol levels relative to DMSO-only controls (Fig. S6i).

Supplemental References

1 Srivastava A, Malik L, Sarkar H, et al. Alignment and mapping methodology influence transcript abundance estimation. Genome Biol 2020;21:239.

2 Srivastava A, Malik L, Smith T, et al. Alevin efficiently estimates accurate gene abundances from dscRNA-seq data. Genome Biol 2019;20:65.

3 Hao Y, Hao S, Andersen-Nissen E, et al. Integrated analysis of multimodal single-cell data. Cell 2021;184:3573-3587.e29.

4 Lai S, Xu Y, Huang W, et al. Mapping Human Hematopoietic Hierarchy at Single Cell Resolution by Microwell-seq. bioRxiv 2017:127217.

5 Lai S, Huang W, Xu Y, et al. Comparative transcriptomic analysis of hematopoietic system between human and mouse by Microwell-seq. Cell Discov 2018;4:34.

6 Chen Y, Lun ATL, Smyth GK. From reads to genes to pathways: differential expression analysis of RNA-Seq experiments using Rsubread and the edgeR quasi-likelihood pipeline 2016. F1000Res; 5:1438.

7 McCarthy DJ, Chen Y, Smyth GK. Differential expression analysis of multifactor RNA-Seq experiments with respect to biological variation. Nucleic Acids Res 2012;40:4288–4297.

8 Robinson MD, McCarthy DJ, Smyth GK. edgeR: a Bioconductor package for differential expression analysis of digital gene expression data. Bioinformatics 2010;26:139–140.

9 Mechanisms and regulation of cholesterol homeostasis. Nat Rev Mol Cell Biol 2020;21:225–245.
